# The Dysregulation of OGT/OGA Cycle Mediates Tau and APP Neuropathology in Down Syndrome

**DOI:** 10.1007/s13311-020-00978-4

**Published:** 2020-11-30

**Authors:** Ilaria Zuliani, Chiara Lanzillotta, Antonella Tramutola, Antonio Francioso, Sara Pagnotta, Eugenio Barone, Marzia Perluigi, Fabio Di Domenico

**Affiliations:** Department of Biochemical Sciences “A. Rossi Fanelli”, Laboratory affiliated to Istituto Pasteur Italia-Fondazione Cenci Bolognetti, Sapienza University of Rome, P.le Aldo Moro 5, 00185 Rome, Italy

**Keywords:** O-GlcNAcylation, Down syndrome, OGT/OGA, APP, tau, autophagy

## Abstract

Protein O-GlcNAcylation is a nutrient-related post-translational modification that, since its discovery some 30 years ago, has been associated with the development of neurodegenerative diseases. As reported in Alzheimer’s disease (AD), flaws in the cerebral glucose uptake translate into reduced hexosamine biosynthetic pathway flux and subsequently lead to aberrant protein O-GlcNAcylation. Notably, the reduction of O-GlcNAcylated proteins involves also tau and APP, thus promoting their aberrant phosphorylation in AD brain and the onset of AD pathological markers. Down syndrome (DS) individuals are characterized by the early development of AD by the age of 60 and, although the two conditions present the same pathological hallmarks and share the alteration of many molecular mechanisms driving brain degeneration, no evidence has been sought on the implication of O-GlcNAcylation in DS pathology. Our study aimed to unravel for the first time the role of protein O-GlcNacylation in DS brain alterations positing the attention of potential trisomy-related mechanisms triggering the aberrant regulation of OGT/OGA cycle. We demonstrate the disruption of O-GlcNAcylation homeostasis, as an effect of altered OGT and OGA regulatory mechanism, and confirm the relevance of O-GlcNAcylation in the appearance of AD hallmarks in the brain of a murine model of DS. Furthermore, we provide evidence for the neuroprotective effects of brain-targeted OGA inhibition. Indeed, the rescue of OGA activity was able to restore protein O-GlcNAcylation, and reduce AD-related hallmarks and decreased protein nitration, possibly as effect of induced autophagy.

**Supplementary Information:**

The online version contains supplementary material available at 10.1007/s13311-020-00978-4.

## Introduction

Down syndrome (DS; Trisomy 21) is the most common chromosomal disorder and the most frequent genetic cause of intellectual disability affecting about 6 million people worldwide [1, 2]. Because of the advances in health care and management of co-occurring illnesses, the life expectancy of people with DS has largely improved [3, 4]. The triplication of genes on chromosome 21 and of their products can alter diverse pathways, including those involved with brain development, metabolism, and neuronal networks [5, 6]. Individuals with DS are also more likely to develop certain pathological conditions, including hypothyroidism, autoimmune diseases, epilepsy, hematological disorders, and Alzheimer-like dementia [7]. The clinical manifestation of Alzheimer-like dementia in DS resembles that occurring in the general population [8, 9], with slight differences in early presentation [10]. Nearly all individuals with full trisomy 21 aged 40 and older are found to have typical signs of Alzheimer’s disease (AD) neuropathology, including extracellular amyloid plaques and intracellular neurofibrillary tangles [11]. The extra copy of amyloid precursor protein (APP) gene on chromosome 21 is associated with a 4- to 5-fold overexpression of APP that leads to an early onset and rapid accumulation of β-amyloid protein (Aβ) with age [9, 12]. Cortical deposits of Aβ 1-42 have even been discovered as early as at 12 years of age [13]. Triplication of specific kinases (e.g., DYRK-1) interacting with APP and tau represents a further link between gene imbalance and neuropathological features DS [14]. Furthermore, brain hypoglycemia and insulin resistance are emerging as common mechanisms of neurodegeneration in DS and AD [15–17]. Several studies using FDG PET at different stages of AD revealed the potential role of reduced glucose uptake in driving neurodegeneration [18–20]. Brain samples from DS and AD demonstrated defects in insulin receptor signaling, decreased levels of brain glucose transporters, decreased activity of enzymes from the Krebs cycle, and a decline in mitochondrial respiratory chain complexes in the brain [5, 16, 21–24].

Recent lines of evidence suggested that the nutrient-related dynamic changes of O-GlcNAcylation might underlie the neuropathological mechanisms observed in AD and DS [25–28]. O-GlcNAcylation is the non-canonical glycosylation of nucleocytoplasmic proteins with a single O-linked N-acetylglucosamine (O-GlcNAc) moiety. The dynamic cycling of O-GlcNAc on proteins is regulated by the concerted actions of two enzymes: the O-GlcNAc transferase (OGT) and a neutral β-hexosaminidase known as O-GlcNAcase (OGA). O-GlcNAcylation occurs widely in all tissues, but heavily in the brain, where OGT and OGA levels were found highly expressed [29–32]. Because O-GlcNAcylation depends on the availability of UDP-GlcNAc, the product of the hexosamine biosynthetic pathway (HBP), and in turn intracellular UDP-GlcNAc level determines OGT activity, O-GlcNAcylation is considered a valuable intracellular sensor of cell metabolic status that can be directly regulated in a nutrient-responsive manner [33]. Notably, O-GlcNAcylation and phosphorylation, by occurring on serine and threonine residues of proteins, are mutually related, allowing cells to modulate a variety of signaling pathways and transcription factors in response to nutrients or stress [34, 35]. Many of the O-GlcNAcylated proteins are directly related to neurodegenerative diseases, including APP, α-synuclein, neurofilaments, tau, and synapsin I [26, 36–40]. Several studies have recently correlated brain hypoglycemia with decreased O-GlcNAcylation supporting their convergence to AD neurodegeneration [23, 26, 28, 41]. The accepted hypothesis is that the general decrease of protein O-GlcNAcylation associated with the specific reduction of APP and tau GlcNAc levels may promote Aβ plaque formation and tau aggregation in the brain, thus contributing to cognitive decline [37, 42–45]. Noteworthy, several studies reported the increase of O-GlcNAc levels in the brain of AD subjects suggesting a more complex crosstalk between O-GlcNAcylation and neurodegenerative processes [42, 46, 47].

Given that DS and AD share similar metabolic alterations and common pathological markers within the brain [8, 16, 48], it is conceivable to suppose a role for aberrant O-GlcNAcylation in driving DS neurodegeneration. The present work discloses, for the first time, the reduction of GlcNAc levels in the hippocampus of a murine model of DS supporting the notion that this phenomenon, by heavily affecting APP and tau post-translational modifications, might have an evident role in the pathological mechanisms mediating the progression of AD-like dementia in DS individuals.

## Materials and Methods

### Animal Model

Experiments were conducted on Ts2Cje mice (Rb(12.Ts171665Dn)2Cje) and correspondent euploid animals (B6EiC3SnF1). Ts2Cje are a well-established murine model of DS characterized by a triple copy of a Robertsonian fusion chromosome carrying the distal end of Chr16 and Chr12. Parental generations were purchased from Jackson Laboratories (Bar Harbor, ME, USA). Mouse colony was raised by repeated crossbreed of Ts2Cje (Ts2) trisomic females with euploid (Eu) males. Since these breeding pairs produce litters containing both trisomic and euploid offspring, resultant progeny was genotyped to determine the presence of the trisomic segment using quantitative PCR, as previously described by Reinoldth et al. [49]. Mice were housed in clear Plexiglas cages (20 × 22 × 20 cm) under standard laboratory conditions with a temperature of 22 ± 2 °C and 70% humidity, a 12-h light/dark cycle and free access to food and water. For the initial longitudinal study, both Ts2 and Eu male and female mice were sacrificed by cervical dislocation at different time points (3, 6, 9, 12, and 18 months of age) and brain areas were collected for preliminary western blot analysis. In order to perform the 6-month-focused analysis, Ts2 and Eu males and females at the selected age (*n* = 6/group) were perfused with saline solution through intracardiac injection. Brain was dissected in halves: one hemisphere was fixed for immunofluorescence analysis while the other section was used for remaining biochemical evaluations. A 2-way ANOVA analysis was performed to exclude the influence of sex (Sup. Tab. 1). All experiments were performed in strict compliance with the Italian National Laws (DL 116/92), and the European Communities Council Directives (86/609/EEC). Experimental protocol was approved by the Italian Ministry of Health (#1183/2016-PR). All efforts were made to minimize the number of animals used in the study and their suffering. All samples were flash-frozen and stored at −  80 °C until utilization.

### Thiamet-G Intranasal Treatment

A pilot study was performed to identify the effective Thiamet-G (TMG; HY-12588, MedChemExpress) dose to use for the intranasal treatment. This preliminary dose-response study was performed on a restricted number of Eu animals (*n* = 3/group; Eu = 3 m) which were respectively administered with vehicle solution (Veh; PBS 1X solution) or 1 𝜇g, 5 𝜇g, 10 𝜇g, 25 𝜇g, and 50 𝜇g of TMG solution. Animals were treated with a single intranasal delivery of 10 𝜇L to each nostril and sacrificed 8 h later through cervical dislocation. Brain areas were collected, and western blot analysis was performed to evaluate treatment efficacy. Our data demonstrated that the intranasal administration of 25 𝜇g of TMG was able to significantly increase the global levels of O-GlcNAcylated proteins (Sup. Fig. 7). Once the effective dose was identified, 6-month-old Ts2Cje and euploid mice were divided into four experimental groups (*n* = 6/group) according to genotype and intranasal treatment received. Based on previously published data on TMG treatment in rodents, which demonstrated that the administration of the drug achieved the peak of increased levels of O-GlcNAc proteins after 8–10 h [50], we opted to treat our animals 2 × day with the effective dose of 25 𝜇g for 5 days, with the aim of achieving a stable elevation of O-GlcNAc proteins for a short period of time. Treatment was well tolerated although a physiological loss of weight was noticed due to prolonged animal manipulation. Indeed, an equal weight loss was observed in TMG-treated mice as well as animals treated with vehicle solution (Table 1). No change in food intake or drinking water consumption was observed. At the end of treatment, animals were euthanized and perfused with sterile PBS through an intracardiac puncture. After sacrifice, brain areas were saved for subsequent analysis.

**Table 1:** Sample characteristics of animals used for aging study, 6-month-old focused analysis and TMG intranasal treatment reporting respective group of treatment, gender, age, experimental use, and average weight before and after TMG treatment

Group		Genotype	*n*	Gender	Age months	Experimental Use				Initial Weight	Final Weight
				(m/f)	(avg ± SD)					g (avg ± SD)	
Aging	3 m	Euploid	6	1/5	3.2 ± 0.5	WB *n* = 6/group					
		Ts2Cje	6	1/5	3.3 ± 0.4						
	6 m	Euploid	6	4/2	6.2 ± 0.4						
		Ts2Cje	6	2/4	6.4 ± 0.5						
	9 m	Euploid	6	1/5	9.4 ± 0.5						
		Ts2Cje	6	3/3	9.1 ± 0.9						
	12 m	Euploid	6	5/1	11.4 ± 0.2						
		Ts2Cje	6	4/2	11.6 ± 0.4						
6-months-old		Euploid	6	4/2	6.2 ± 0.4	WB *n* = 6/group	qRT-PCR *n* = 5/group	IP *n* = 4/group			
		Ts2Cje	6	2/4	6.4 ± 0.5						
		Euploid	3	2/1	5.3 ± 1.1	Immunofluorescence *n* = 3/group					
		Ts2Cje	3	2/1	5.6 ± 0.8						
		Euploid	5	3/2	5.5 ± 0.6	OGA and GFAT1 Assays *n* = 5/group					
		Ts2Cje	5	2/3	5.3 ± 0.6						
Treatment	Veh	Euploid	6	3/3	6.7 ± 0.8	WB SB ELISA *n* = 6/group	qRT-PCR *n* = 5/group	OGA Assay *n* = 5/group	IP *n* = 4/group	38.6 ± 9.8	37.9 ± 9.4
		Ts2Cje	6	3/3	6.7 ± 0.8					33.3 ± 6.0	32.5 ± 5.0
	TMG	Euploid	6	3/3	6.8 ± 0.4					34.4 ± 8.8	33.9 ± 8.1
		Ts2Cje	6	4/2	6.6 ± 0.5					31.7 ± 4.7	30.0 ± 5.6

### Immunofluorescence

Entire brains from 6-month-old Ts2Cje mice and corresponding euploids were fixed in a 4% formaldehyde aqueous solution for 24 h at 4 °C. Fixed brains were then cryoprotected for the next 48 h at 4 °C with a solution containing 20% of sucrose and 0.02% of NaN_3_.

Brains were frozen on a temperature-controlled freezing stage, coronal sectioned (20 μm) on a sliding cryostat (Leica Biosystems, Wetzlar, Germany), and stored in a solution of PBS containing 0.02% NaN_3_ at 4 °C until utilization. Brain sections were mounted on glass slide. Once dried, brain sections underwent to a heat-induced antigen retrieval step in a 10 mM EDTA solution, pH = 6.0, for 20 min at 55 °C [51]. After 4 washes with filtered PBS, sections were blocked with a solution containing 10% normal goat serum and 0.2% Triton X-100 in filtered PBS. Slides were then incubated 24 h at 4 °C with following antibodies: GFAP (1:500; anti-rabbit; 840,001, BioLegend), IBA1 (1:250; anti-rabbit; GTX100042, GeneTex), NeuN-1 (1:500; anti-rabbit, 702,022, Invitrogen Thermo Fisher Scientific), O-GlcNAc CTD110.6 (1:100; anti-mouse, SC-59623, Santa Cruz Biotechnology), O-GlcNAc RL2 (1:50; anti-mouse, #MABS157, Sigma-Aldrich). Slides were then washed with filtered PBS and incubated with Alexa Fluor − 488 nm and − 594 nm secondary antibodies (1:1500; A11029, A11034, Invitrogen Thermo Fisher Scientific) for 1.5 h at room temperature. Tissues were then stained with Sudan black (0.1% Sudan Black B in 70% ethanol; 199,664, Sigma-Aldrich) to block auto fluorescence inherent to the sample. Slides were then washed, incubated with DAPI (10 mg/mL; IS-7712, Immunological Sciences) for 1 min and washed again. One slide per group was stained without primary antibodies to establish nonspecific background signal. At the end, cover slip glasses were placed using a drop of Fluoromount aqueous mounting medium (F4680, Sigma-Aldrich) and glasses were kept at room temperature to dry. All slides were imaged using Zeiss AXio (Carl Zeiss, Oberkochen, Germany). All immunolabeling acquisition intensities, field sizes, and microscopy settings were kept consistent across images. Images were analyzed using ImageJ. Image montages for figures were collated in Illustrator and Photoshop Cs6 (Adobe System) software programs and were based upon brain images that most closely approximated the group means.

### Western Blot

All mice samples used for longitudinal and coming from intranasal TMG treatment were homogenized following the same procedure. The hippocampus region was thawed in RIPA buffer (pH 7.4) containing 50 mM Tris-HCl (pH 7.4), 150 mM NaCl, 1% NP-40, 0.25% sodium deoxycholate,1 mM EDTA, 0.1% SDS, protease inhibitor cocktail (1:100; 539132, Millipore), phosphatase inhibitor cocktail (1:100; P5726, Sigma-Aldrich), PUGNAc (OGA inhibitor, 100 𝜇M; A7229, Sigma-Aldrich), and benzyl-2-acetamido-2-galactopyranose (OGT inhibitor, 2 mM; B4894, Sigma-Aldrich). Brains were homogenized by 20 strokes of a Wheaton tissue homogenizer, sonicated, and centrifuged at 14000 rpm for 40 min at 4 °C to remove debris. Supernatant was collected and total protein concentration was determined by the BCA method (Pierce™ BCA Protein Assay Kit, 23227, Thermo Fisher Scientific, according to the manufacturer’s instructions. For western blot analysis, 15 𝜇g of proteins was separated via SDS-PAGE using Criterion™ TGX Stain-Free™ precast gel (Bio-Rad) and transferred to a nitrocellulose membrane by Trans-Blot Turbo Transfer System (Bio-Rad). The blot was imaged by ChemiDoc MP imaging system (Bio-Rad) using the Stain-Free Blot settings. Protein total load captured by Stain-Free technology was later used for total protein normalization. Following, nitrocellulose membrane was blocked using 3% BSA (bovine serum albumin; 9048-46-8, SERVA) or Milk 5% (skim milk powder; 42,590, SERVA) in 1 × Tris-buffered saline (TBS; #1706435, Bio-Rad) containing 0.01% Tween20 and incubated overnight at 4 °C with the following primary antibodies: α-CTF and β-CTF (1:5000; SAB5200113, Sigma-Aldrich), p^Thr172^AMPK (1:1000; GTX52341, GeneTex), AMPKα1/2 (1:500; SC-74461, Santa Cruz Biotechnology), APP (1:5000; SAB5200113, Sigma-Aldrich), p^Thr642^AS160 (1:1000; GTX55118, GeneTex), AS160 (1:500; MA514840, Invitrogen Thermo Fisher Scientific), AT8 (1:1000; MN1020, Invitrogen Thermo Fisher Scientific), Atg7 (1:1000; SC-376212, Santa Cruz Biotechnology), Atg5-12 (1:1000; SC-133158, Santa Cruz Biotechnology), BDNF (1:500; SC-546, Santa Cruz Biotechnology), Beclin-1 (1:1000; 3738, Cell Signaling Technology), GFAP (1:5000; 840001, BioLegend), p^Ser243^GFAT1 (1:1000; S343C, MRC-PPU), GFAT1 (1:1000; 28121, IBL), GLUT1 (1:500; ab40084, Abcam), GLUT3 (1:500; SC-74497, Santa Cruz Biotechnology), GLUT4 (1:500; SC-53566, Santa Cruz Biotechnology), p^Ser9^GSK3β (1:1000; 5558, Cell Signaling Technology), p^Tyr216^GSK3β (1:500; SC-81496, Santa Cruz Biotechnology), GSK3β (1:500; SC-377213, Santa Cruz Biotechnology), IBA1 (1:1000; GTX100042, GeneTex), p^Tyr1146/1150/1151^IR (1:1000; GTX25681, GeneTex), IR (1:1000; 3020, Cell Signaling), p^Tyr612^IRS1 (1:1000; GTX24868, GeneTex), p^Ser636^IRS1 (1:1000; GTX32400, GeneTex), LC3 I-II (1:1000; NB1002220, Novus Biologicals), mTOR (1:1000; 2983, Cell Signaling Technology), p^Ser2448^mTOR (1:1000; 5536, Cell Signaling Technology), OGA (1:1000; SAB-4200267, Sigma-Aldrich), O-GlcNAc CTD110.6 (1:500; SC-59623, Santa Cruz Biotechnology), O-GlcNAc RL2 (1:1000; MABS157, Sigma-Aldrich), OGT (1:500; SC-74546 Santa Cruz Biotechnology), PSD95 (1:1000; 3450, Cell Signaling Technology), pSer/Thr (1:5000; ab17464, Abcam), Syntaxin 1A (1:1000; Ab1453, Abcam), SQSTM1 (1:1000; SC-28359, Santa Cruz Biotechnology), p^Ser404^tau (1:1000; ab92676, Abcam), tau (1:1000; orb46243, Biorybt). The following day, all membranes were washed with 1 × TBS containing 0.01% Tween20 and incubated at room temperature for 1 h with respective horseradish peroxidase–conjugated secondary antibodies: anti-rabbit (1:10000; L005661, Bio-Rad Laboratories), anti-mouse (1:10000; L005662, Bio-Rad Laboratories), anti-sheep, (1:3000; A3415, Sigma-Aldrich). As necessary, enhanced sensitivity was obtained using secondary antibodies able to detect only native IgG (1:200; TidyBlot, #STAR209, Bio-Rad Laboratories. 1:1000; TrueBlot, 18-8817-30, Rockland Immunochemicals). Blots were then imaged via the ChemiDoc MP imaging system using Chemiluminescence settings. Subsequent determination of relative abundance via total protein normalization was calculated using Image Lab 6.1 software (Bio-Rad Laboratories).

### Slot Blot

For the analysis of total protein-bound 4-hydroxy-2-nonenals (HNE adducts) and 3-nitrotyrosine (3-NT) levels, 3 μl of hippocampus homogenate from Ts2Cje and Eu both treated with TMG and vehicle (*n* = 6/group) was incubated with 6 μl of Laemmli Buffer (0.125 M Tris base pH = 6.8, 4% (v/v) SDS, and 20% (v/v) glycerol). The resulting samples (250 ng/well) were loaded under vacuum onto a nitrocellulose membrane using a slot blot apparatus. Membranes were blocked for 1 h at room temperature with 3% of bovine serum albumin in TBS solution containing 0.01% Tween 20 and incubated at room temperature for 2 h with the corresponding primary antibodies: 3-NT (1:1000; N5538, Sigma-Aldrich) and HNE polyclonal antibody (1:2000; NB100–63093, Novus Biologicals). Membranes were then washed three times with TBS solution containing 0.01% Tween 20 and incubated for 1 h at room temperature with respective alkaline phosphatase secondary antibodies from Sigma-Aldrich: anti-mouse (A1293; 1:3000) and anti-goat (A4187; 1:3000). Membranes were later washed three times in TBS solution containing 0.01% Tween 20 and developed with Sigma Fast BCIP/NBT (5-bromo-4-chloro-3-indolyl phosphate/nitro blue tetrazolium substrate). Blots were dried, acquired with Chemi-Doc MP imaging system, and analyzed using Image Lab 6.1 software (Bio-Rad Laboratories).

### Immunoprecipitation

#### For OGT

Sepharose beads were used to immunoprecipitate OGT (EZView Red Protein G Beads, Sigma-Aldrich) according to the manufacturer’s instructions. Briefly, different sample sets (100 μg of proteins; *n* = 4/group) were incubated overnight at 4 °C with the primary antibody for OGT (1:100; SC-74546 Santa Cruz Biotechnology) in IP buffer containing 10 mM Tris (pH = 7.6), 140 mM NaCl, 0.5% NP40, phosphatase inhibitor cocktail (1:100; P5726, Sigma-Aldrich), PUGNAc (OGA inhibitor, 100 𝜇M; A7229, Sigma-Aldrich), and benzyl-2-acetamido-2-galactopyranose (OGT inhibitor, 2 mM; B4894, Sigma-Aldrich). The following day, all samples were incubated with 20 𝜇L of protein G beads (EZView Red Protein G Beads, E3403, Sigma-Aldrich) for 2 h at room temperature and then washed three times with RIA buffer containing 10 mM Tris (pH = 7.6), 140 mM NaCl, 1% NP40. Afterwards, standard western blot procedure was performed. Resulting blots were incubated overnight at 4 °C with the primary antibodies O-GlcNAc CTD110.6 (1:500; SC-59623, Santa Cruz Biotechnology), O-GlcNAc RL2 (1:1000; MABS157, Sigma-Aldrich), OGT (1:500; SC-74546, Santa Cruz Biotechnology), and pSer/Thr (1:5000; ab17464, Abcam), and detected by the horseradish peroxidase-conjugated secondary antibodies: anti-mouse (1:10000; L005662, Bio-Rad Laboratories) and anti-rabbit (1:10000; L005661, Bio-Rad Laboratories). IP results were normalized on the total amount of OGT and analyzed following the same procedures used for western blot.

#### For APP or Tau

Magnetic beads were used to immunoprecipitate APP and tau (SureBeads™ Protein G Magnetic Beads; 1614023, Bio-Rad Laboratories), according to the manufacturer’s instructions. Briefly, 100 μL of magnetic beads was magnetized using specific tube-magnetic rack and washed three times with 1 × PBS containing 0.1% Tween20. Primary antibody for APP (1:100; SAB5200113, Sigma-Aldrich) or tau (1:50; orb46243, Biorybt) was incubated with magnetic beads for 30 min at room temperature. After three washes, 100 μg of proteins (*n* = 4/group) for each sample was incubated for 90 min at room temperature. After an additional three washes, standard western blot procedure was performed for APP and tau IP. Resulting blots were incubated overnight at 4 °C with the primary antibodies APP (1:5000; SAB5200113, Sigma-Aldrich), tau (1:1000; orb46243, Biorybt), O-GlcNAc CTD110.6 (1:500; SC-59623, Santa Cruz Biotechnology), O-GlcNAc RL2 (1:1000; MABS157, Sigma-Aldrich), and pSer/Thr (1:5000; ab17464, Abcam) and detected by the horseradish peroxidase–conjugated secondary antibodies anti-mouse (1:10000; L005662, Bio-Rad Laboratories), anti-rabbit (1:10000; L005661, Bio-Rad Laboratories), and horseradish peroxidase–conjugated secondary antibodies able to detect only native IgG (1:200; TidyBlot, #STAR209, Bio-Rad Laboratories; 1:1000; TrueBlot, 18-8817-30, Rockland Immunochemicals). IP results were normalized on the total amount of APP or tau and analyzed following the same procedures used for western blot.

### OGA Assay

OGA enzymatic activity was measured using the synthetic substrate *p-*nitrophenyl N-acetyl-β-d-glucosaminide (pNP-GlcNAc) as described by Zachara and colleagues [52]. All samples used for 6-month-focused analysis and intranasal TMG treatment were processed using the same procedure. Briefly, 15 mg of hippocampus was thawed in RIPA buffer (pH = 7.4) containing 50 mM Tris (pH = 7.4), 50 mM NaCl, 1% NP-40, 0.25% sodium deoxycholate,1 mM EDTA, 0.1% SDS, protease inhibitor cocktail (1:100; 539132, Millipore), and phosphatase inhibitor cocktail (1:100; P5726, Sigma-Aldrich). Brains were homogenized by 20 strokes of a Wheaton tissue homogenizer, sonicated and centrifuged at 14,000 rpm for 40 min at 4 °C to remove debris. Supernatant was collected, desalted using Zeba™ Spin Desalting Columns (89882; Thermo Fisher Scientific), and protein concentration was determined by the BCA method (Pierce™ BCA Protein Assay Kit, 23227, Thermo Fisher Scientific) according to the manufacturer’s instructions. Samples (150 μg of proteins) were incubated with activity assay buffer containing 2 mM pNP-GlcNAc, 50 mM sodium cacodylate (pH = 6.4), 50 mM N-acetylgalactosamine, and 0.3% BSA at 37 °C for 2 h. Reaction was stopped by the addition of 500 mM Na_2_CO_3_ and absorbance was measured at 405 nm (Multiskan EX, Thermo Labsystems). OGA activity is reported as enzyme activity units where 1 U catalyzes the release of 1 μmol pNP/min from pNP-GlcNAc. OGA activity for each group was normalized on corresponding protein expression levels.

### GFAT1 Assay

GFAT1 enzymatic activity was performed adapting a procedure developed by McClain and colleagues [53]. GFAT1 activity for 6-month-focused analysis was assessed through the measuring of its enzymatic product glucosamine 6-phosphate (GlcN6P). Briefly, 15 mg of hippocampus (*n* = 6/group) was thawed in 80 μL lysis buffer (pH = 7.5) containing 100 mM KCl, 1 mM EDTA, 50 mM Na_3_PO_4_, protease inhibitor cocktail (1:100; 539132, Millipore), and phosphatase inhibitor cocktail (1:100; P5726, Sigma-Aldrich). Brains were homogenized by 20 strokes of a Wheaton tissue homogenizer, sonicated and centrifuged at 14000 rpm for 40 min at 4 °C to remove debris. Supernatant was collected and total protein concentration was determined by the BCA method (Pierce™ BCA Protein Assay Kit, 23227, Thermo Fisher Scientific) according to the manufacturer’s instructions. Samples (240 μg of proteins) were incubated with activity assay buffer containing 1 mM EDTA, 1 mM DTT, 40 mM NaHPO_4_ (pH = 7.4), 12 mM fructose 6-phosphate, and 12 mM l-glutamine at 37 °C for 45 min. Reaction was stopped by the addition of PCA 1 M (1:2) to induce protein precipitation. Samples were then incubated 10 min on ice and centrifuged at 16000*g* 4 °C for 10 min. Supernatant was extracted with chloroform (1:2) and 100 μL of the aqueous phase was collected for HPLC analysis. GlcN6P generated during the reaction was detected by derivatization of the sample with 2 volumes of *o*-phthalaldehyde (OPA) reagent (100 μL of 10 mg/mL OPA in EtOH, 900 μL sodium borate 100 mM pH = 9.7, and 2 μL 3-mercaptopropionic acid). Reaction was incubated for 10 min at room temperature protected from light, and sample was diluted 1:1000 in the mobile phase for HPLC detection. Chromatographic separation was performed using an isocratic elution; the mobile phase was composed by Na_3_PO_4_ 15 mM, pH = 7.2 (phase A) and acetonitrile (phase B) (90:10). A Symmetry C18 column (300 Å, 5 μm, 4.6 mm × 250 mm, 1/pk, Waters Corporation) was used for separation. Fluorescence of the sample eluent (λ = 340/450) was analyzed using a fluorescent detector (RF-551, Shimadzu) and the peak area was integrated using dedicated software (Empower 2, Waters Corporation). OPA-derivatized GlcN6P standards (G5509, Sigma-Aldrich) were run separately to determine the retention time (1.8 s) and to generate a standard curve to correlate area to activity. The correlation coefficient between the concentration of GlcN6P standards and the area under the GlcN6P peak was 0.999. Activity is expressed as units/milligram of protein where 1 U represents the generation of 1 pmol of GlcN6P/min. GFAT1 activity for each group was normalized on corresponding protein expression levels.

### RNA Extraction and Quantitative Real-Time RT PCR

RNA was extracted from the hippocampus of Ts2Cje and Eu treated both with TMG and vehicle (*n* = 6/group) using Tissue Total RNA Kit according to the manufacturer’s instructions (Abcam). RNA was quantified using the Biospec Nano spectrophotometer (Shimadzu, Columbia, MD, USA), and RNA was reverse transcribed using the cDNA High-Capacity kit (Applied Biosystems, Foster City, CA, USA), including reverse transcriptase, random primers, and buffer according to the manufacturer’s instructions. The cDNA was produced through a series of heating and annealing cycles in the MultiGene OPTIMAX 96-well thermocycler (LabNet International, Edison, NJ, USA). Real-time PCR (Q-PCR) was carried out using the following cycling conditions: 35 cycles of denaturation at 95 °C for 20 s; annealing and extension at 60 °C for 20 s, using the SensiFAST™ SYBR® No-ROX Kit (Bioline, London, UK). PCR reactions were carried out in a 20-μl reaction volume in a CFX Connect Real Time PCR machine (Bio-Rad Laboratories). Primers used for the evaluation of gene expression were designed as follows: GADPH (Fw: ACAGTCCATGCCATCACTGCC; Rv: GCCTGCTTCACCACCTTCTTG), OGA (Fw: TGGAAGACCTTGGGTTATGG; Rv: TGCTCAGCTTCTTCCACTGA), OGT (Fw: CTGTCACCCTTGACCCAAAT; Rv: ACGAAGATAAGCTGCCACAG). Relative mRNA concentrations were calculated from the take-off point of reactions (threshold cycle, Ct) using the comparative quantification method performed by Bio-Rad software and based upon the ∆∆Ct method. Ct values for GAPDH expression served as a normalizing signal [54].

### Aβ42 Elisa

Mouse Aβ 1-42 ELISA Kit (KMB3441; Invitrogen Thermo Fisher Scientific) was used to determine the levels of amyloid β 1-42 peptide in Ts2 and Eu mice treated with Veh or TMG (*n* = 6/group). Briefly, ~ 10 mg of hippocampus was thawed in ice-cold DEA buffer (10 μL/mg tissue; 0.2% diethanolamine in 50 mM NaCl) with protease inhibitor cocktail (1:100; 539132, Millipore). After centrifugation (15,000 rpm 90 min 4 °C), supernatant was retained as Aβ soluble fraction. Aβ1-42 was then measured according to the manufacturer’s instructions. Curve-fitting was obtained by Graph Pad Prism 8.0 software (GraphPad, La Jolla, CA, USA).

### Statistical Analysis

Statistical analyses were performed using Student *t* test for the evaluation of differences between two groups and a non-parametric 1-way ANOVA with post hoc Bonferroni *t* test for the evaluation of differences between more than two groups. To determine the influence of genotype (Ts2Cje and euploids), treatment (TMG and vehicle), or sex (male and female), we performed a 2-way ANOVA analysis. Data are expressed as mean ± SEM per group. All statistical analyses were performed using Graph Pad Prism 8.0 software (GraphPad, La Jolla, CA, USA).

## Results

### Ts2Cje Mice Show an Aberrant and Tissue-Specific O-GlcNAcylation Profile at 6 Months of Age

**Fig. 1:**
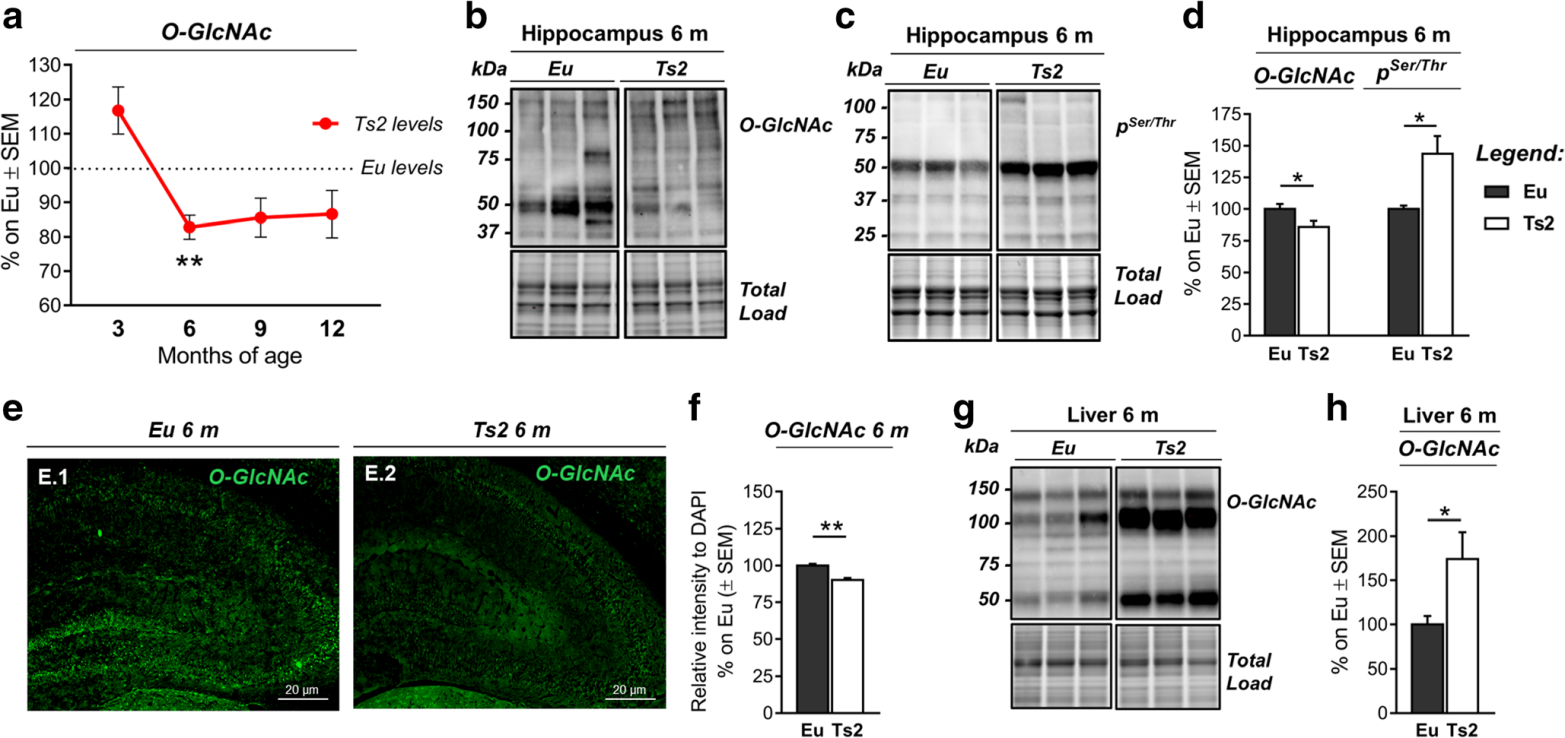
Early alteration of O-GlcNAcylation and phosphorylation profile in Ts2Cje mice. (A) Longitudinal study of the O-GlcNAcylation profile in the hippocampus of differently aged Ts2Cje mice compared to respective euploids. A premature impairment of protein O-GlcNAcylation was observed in 6-month-old Ts2 mice compared to aged-matched euploids. A pronounced dropping of O-GlcNAcylated protein levels was detected also at 9 and 12 months of age. Representative blots are reported in Fig. S1. (B–D): O-GlcNAcylation/phosphorylation profile in 6-month Ts2Cje mice hippocampus compared to respective euploids. The reduction of protein O-GlcNAcylation in the hippocampus of 6-month-old Ts2 mice was in line with a mutual inverse increase in the global phosphorylation of serine and threonine residues compared to aged-matched controls. Representative blots are reported in (B) and (C). (E–F) Immunofluorescence analysis of O-GlcNAcylated proteins in the hippocampus of 6-month-old Ts2Cje and respective euploid mice. A diffuse impairment of O-GlcNAcylated proteins was detected in the entire hippocampal area from Ts2 mice compared to aged-matched euploids. Relative intensity quantification is reported in (F). (G–H) O-GlcNAcylation/phosphorylation profile in 6-month Ts2Cje mice liver compared to respective euploids. Increased levels of O-GlcNAcylated proteins were observed in the liver of Ts2 mice compared to euploid animals of the same age, confirming a global imbalance of O-GlcNAcylation homeostasis. Representative blot is reported in (G). Number of animals for each condition was as follows: *n* = 6/group for western blot and *n* = 3/group for immunofluorescence staining. All bar charts reported in (A), (D), (F), and (H) show mean ± SEM. *p < 0.05, **p < 0.01, using Student’s *t* test

In the last few years, strong interest has been focused on understanding the role of protein O-GlcNAcylation in the development of AD-associated pathological features [26, 28, 36, 38, 55–57]. We took advantage of the Ts2Cje (Ts2) mice to investigate the O-GlcNAcylation profile and its relevance in DS neurodegeneration. To begin, we conducted an age-dependent study with the aim of assessing putative changes of the total levels of O-GlcNAcylated proteins in the hippocampus from 3-, 6-, 9-, and 12-month-old Ts2 compared with aged-matched euploids (Eu). We observed, in Ts2 mice, a premature reduction of O-GlcNAcylated proteins as early as at 6 months of age. Indeed, we found a trend of increase of total O-GlcNAc levels in 3-month-old Ts2 hippocampus, compared to aged-matched euploids, but a sudden significant switch is shown at 6 months of age (Fig. 1(A); **p < 0.01, Eu vs Ts2: − 18%). A trend of reduced O-GlcNAc levels persisted also in Ts2 hippocampus at 9 months of age and 12 months of age compared to respective euploids, suggesting the reduction of O-GlcNAc levels during aging. Accordingly, we observed by immunofluorescence microscopy a diffuse reduction of O-GlcNAcylated proteins in the entire hippocampus area of Ts2 mice at 6 months of age compared to respective euploids (Fig. 1(E–F); **p < 0.01, Eu vs Ts2: − 10%).

Considering the significant alteration of protein O-GlcNAcylation in 6-month-old DS mice, we focused our following experiments on this age group. To investigate the interplay between O-GlcNAcylation and O-phosphorylation on serine-threonine residues [35, 38, 45, 58, 59], we evaluated the levels of total protein phosphorylation on these residues only. As postulated, we detected a significant increase of total protein phosphorylation in Ts2 hippocampus compared to respective euploids at 6 months of age (Fig. 1(C–D); *p < 0.05, Eu vs Ts2: + 44%). Several studies supported that in AD and metabolic diseases, the reduction of protein O-GlcNAcylation is a brain-specific effect associated with reduced glucose uptake, altered HBP flux, and/or aberrant phosphorylation process [23, 26, 41, 60, 61]. In contrast, peripheral organs often demonstrate an increased trend of O-GlcNAcylated proteins which correlates hyperglycemia and contributes to impaired insulin signaling and glucose toxicity [62–65]. Our analysis of liver samples from 6-month Ts2 mice compared to relative euploids demonstrated a significant increase of global protein O-GlcNAcylation (Fig. 1(G–H); *p < 0.05, Eu vs Ts2: + 74%), thus confirming the tissue specificity of this PTMs. Further, the early presence of alterations both in the CNS and in the liver of 6-month-old Ts2Cje mice suggests that aberrant protein O-GlcNAcylation contributes to DS pathogenesis promoting, in different organs, peculiar mechanisms of disease development.

### The Reduction of O-GlcNAcylated Proteins in the Hippocampus of 6-Month-Old Ts2Cje Mice Is Area and Cell-Type Specific

**Fig. 2:**
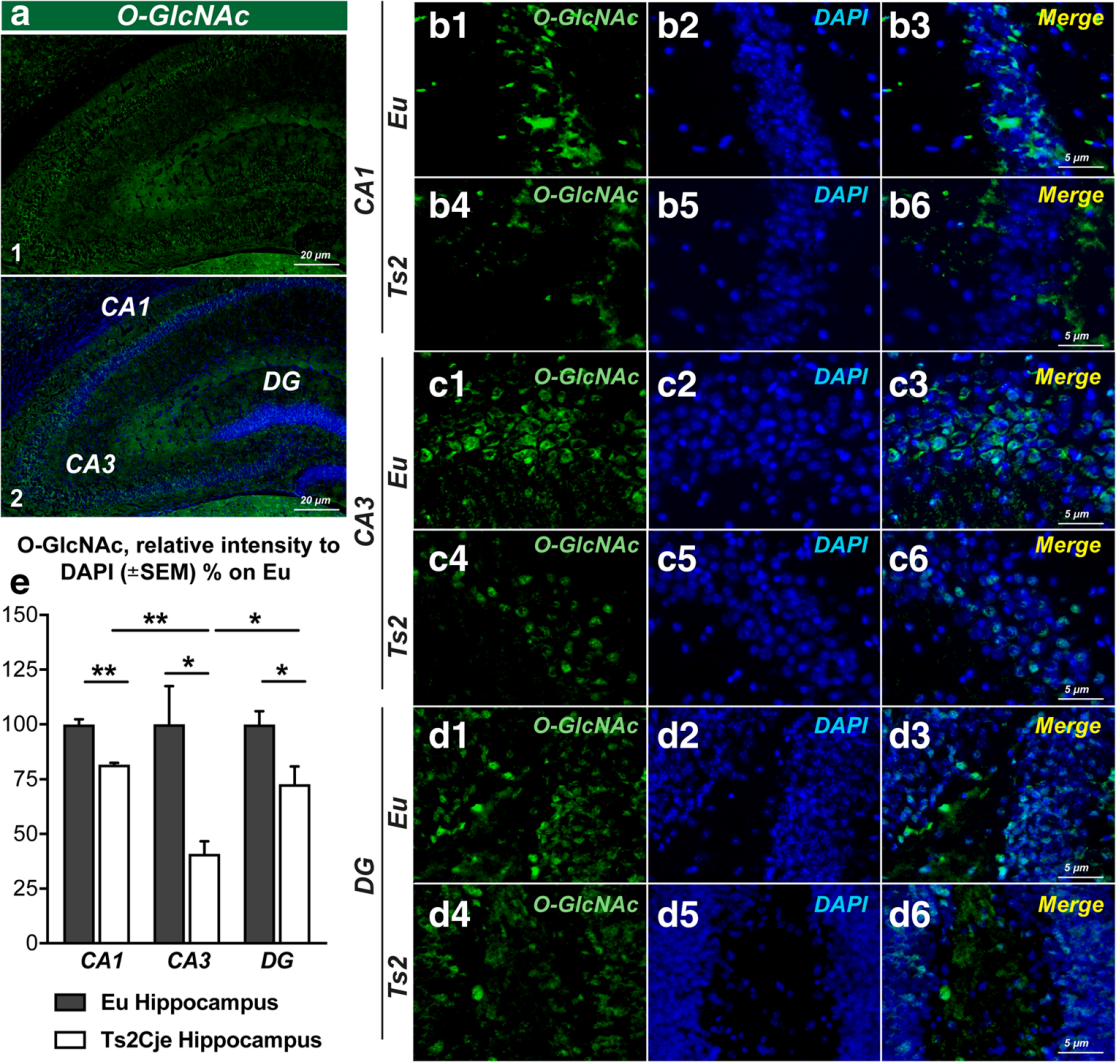
The reduction of O-GlcNAcylated proteins is area specific in the hippocampus of Ts2Cje mice. (A1–2) O-GlcNAc staining of the entire hippocampus from 6-month-old Ts2Cje mice. (B-6) O-GlcNAc staining of the CA1 area of the hippocampus from Ts2 and respective euploids. A significant impairment of global protein O-GlcNAcylation in the CA1 area of Ts2 mice (B1–3) was observed in comparison with to the fluorescent signal of the same area from aged-matched euploids (B4–6); O-GlcNAc (green); DAPI (blue). (C1–6) O-GlcNAc staining of the CA3 area of the hippocampus from Ts2 and respective euploids. Ts2 mice showed a massive reduction of global protein O-GlcNAcylation in the CA3 hippocampal area (C1–3) compared to respective euploids (C4–6); O-GlcNAc (green); DAPI (blue). (D1–6) O-GlcNAc staining of the DG area of the hippocampus from Ts2 and respective euploids. A similar impairment was observed in the dentate gyrus from Ts2 mice (D1–3) compared to the same brain region from euploid animals (D4–6); O-GlcNAc (green); DAPI (blue). (E) Related quantification of O-GlcNAc fluorescence intensity normalized on DAPI signal is reported for each hippocampal region from Ts2 and Eu animals. Number of animals for each condition was as follows: *n* = 3/group for immunofluorescence staining. All bar charts reported in (E) show mean ± SEM. *p < 0.05, **p < 0.01, using Student’s *t* test

**Fig. 3:**
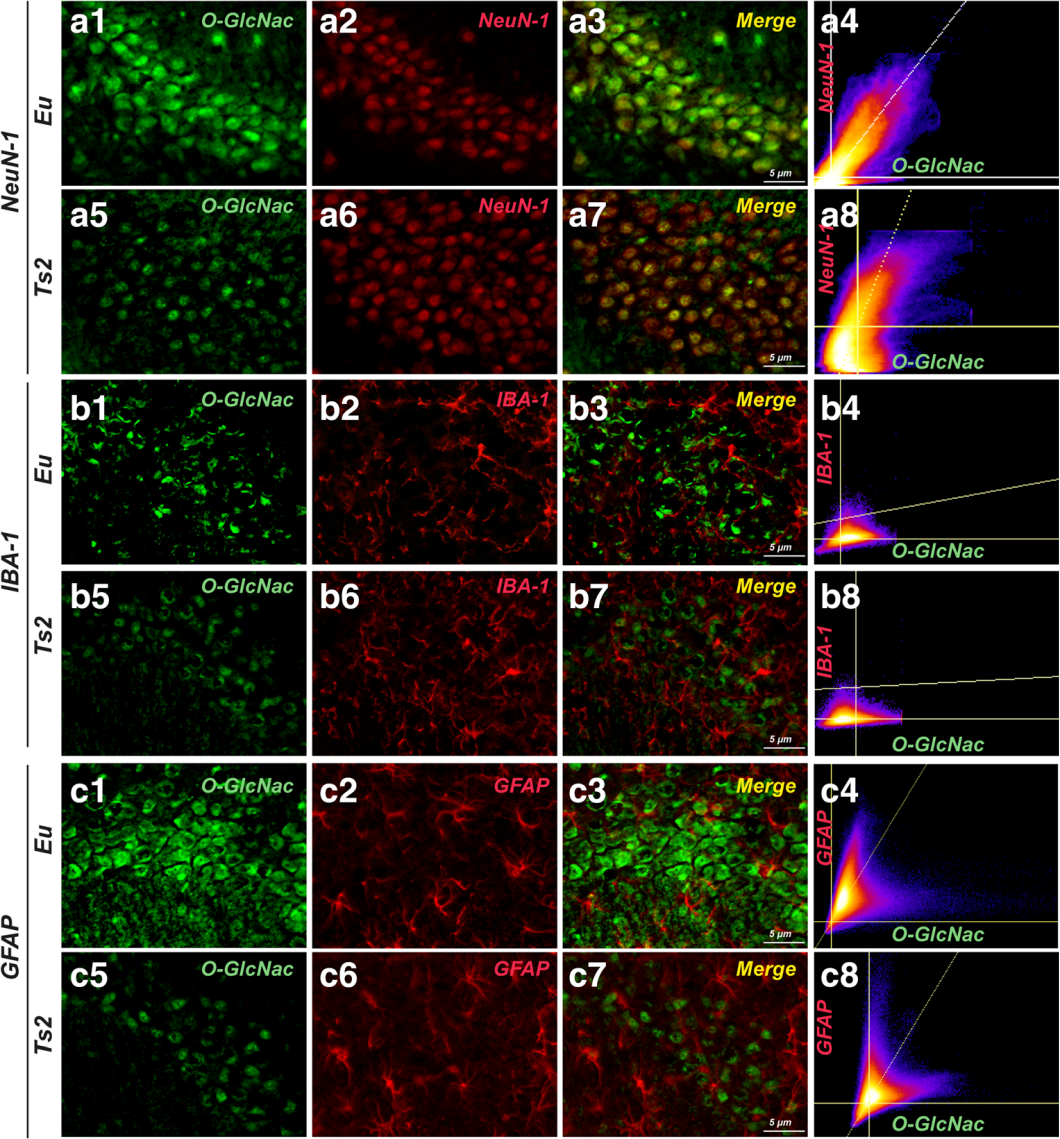
The reduction of protein O-GlcNAcylated proteins is cell-type specific in the hippocampus of Ts2Cje mice. (A1–8) O-GlcNAc co-localization with the neuronal marker NeuN-1 in the CA3 hippocampal area from Ts2 and respective euploid mice. O-GlcNAc signal seems to broadly overlap with neuronal marker NeuN-1 in both CA3 area from euploid animals (A1–4) and Ts2 mice (A5–8). Co-localization graphs are reported for both Eu (A4) and Ts2 (A8) mice; O-GlcNAc (green); NeuN-1 (red); co-localization (yellow). (B1–8) O-GlcNAc co-localization with glial marker IBA-1 in the CA3 hippocampal area from Ts2 and respective euploid mice. Partial co-localization was observed between O-GlcNAcylated proteins and fluorescent signal from the glial marker IBA-1 in Ts2 CA3 subregion (B1–4) and a comparable result was obtained in the same region of euploid animals (B5–8). Co-localization graphs are reported for both Eu (B4) and Ts2 (B8) mice; O-GlcNAc (green); IBA-1 (red); co-localization (yellow). (C1–8) O-GlcNAc co-localization with the astrocytic marker GFAP in the CA3 hippocampal area from Ts2 and respective euploid mice. Consistent co-localization of O-GlcNAc signal and GFAP was detected in the CA3 area of euploid animals (C1–4), while apparently no signal has been identified in the same area of Ts2 hippocampus (C5–8). Co-localization graphs are reported for both Eu (C4) and Ts2 (C8) mice; O-GlcNAc (green); GFAP (red); co-localization (yellow). Number of animals for each condition was as follows: *n* = 3/group for immunofluorescence staining

Although almost all cerebral tissues contain O-GlcNAcylated proteins, O-GlcNAc and OGT are particularly abundant in the hippocampal region [29, 31, 55, 66]. Furthermore, O-GlcNAcylation plays a role in regulating hippocampal synaptic transmission and plasticity, thus influencing learning and memory processes [67, 68]. Considering the relevance of protein O-GlcNAcylation in this brain area, we further examined the distribution of O-GlcNAcylated proteins in different subregions of Ts2 hippocampus at 6 months of age. As expected, Ts2 mice showed a general impairment of protein O-GlcNAcylation compared to respective euploids in each of the hippocampal subregions analyzed (Fig. 2(A1–2)). In detail, a relevant reduction of O-GlcNAc fluorescent signal was observed in the CA1 area (Fig. 2(B1–6, E); **p < 0.01, Eu vs Ts2: − 20%), in the CA3 area (Fig. 2(C1–6, E); *p < 0.05, Eu vs Ts2: − 70%), and in the dentate gyrus (DG) region (Fig. 2(D1–6, E); *p < 0.05, Eu vs Ts2: − 30%) of the hippocampus from Ts2 mice compared to the respective euploid mice. In addition, Ts2 mice showed a different distribution of O-GlcNAcylated protein reductions, with a higher decrease O-GlcNAc levels in the CA3 subregion compared to both the DG area and the CA1 area (Fig. 2(E)). In the second instance, we evaluated cell-type distribution of O-GlcNAcylated proteins in the CA3 subregion, which has proved to be the area most affected by the reduction of O-GlcNAc fluorescence intensity in Ts2 mice. We determined that protein O-GlcNAcylation occurs primarily in neurons in Ts2Cje (Fig. 3(A5–8)). Indeed, co-localization analysis showed significant O-GlcNAc signal (green) that overlaps with the neuronal marker NeuN-1 (red) (Fig. 3(A5–8)), while partial or no overlap between O-GlcNAc and microglia (IBA-1) or astrocytes (GFAP) occurs (Fig. 3(B5–8, C5–8) respectively) in the CA3 of 6-month-old Ts2 mice. Costes’ and Manders’ coefficient (M1 and M2) analyses of co-localization showed that 90% of the O-GlcNAc signal co-localized with neurons [69]. In Eu mice, co-localization analysis demonstrated a strong correlation as well with NeuN-1 and O-GlcNAc that, although with lower values, persists also in microglia and astrocytes. These results suggest a cell-type specific impairment of O-GlcNAcylated proteins in Ts2Cje at 6 months of age and a consistent reduction of protein O-GlcNAcylation in neurons and astrocytes. We note that the analysis of GFAP and IBA-1 markers by immunochemical methods demonstrated no significant changes in Ts2 compared to Eu mice, thus supporting no astrocytosis or microgliosis at 6 months of age (Sup. Fig 2) [70].

### Reduced O-GlcNAcylation Results from the Dysregulation of OGT/OGA Cycle

**Fig. 4:**
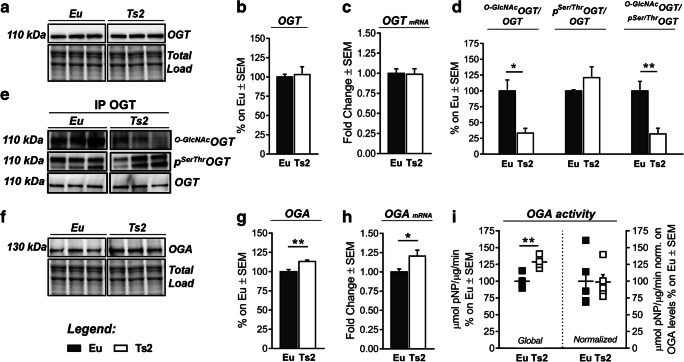
Reduced O-GlcNAcylation rely on aberrant OGT/OGA cycling. (A–C) Analysis of OGT protein levels and transcript in Ts2 mice compared to respective euploids. OGT showed no alteration neither in protein expression nor in mRNA levels in Ts2 hippocampus compared to the control group. Representative blot is reported in (A). (D–E) Evaluation of OGT’s PTMs by immunoprecipitation analysis. A significant reduction in ^O-GlcNAc^OGT/OGT levels together with a trend of increase in its p^Ser/Thr^OGT/OGT levels was observed in Ts2 mice compared to the respective euploid group. Representative blots are reported in (E). (F–H) Analysis of OGA protein levels and transcript in Ts2 mice compared to respective euploids. Both OGA protein and mRNA levels were found significantly increased Ts2 mice in comparison to the respective control group. Representative blot is reported in (F). (I) OGA activity assay. Global OGA activity is significantly increased in Ts2 mice compared to the respective control group. However, the enzyme-specific hydrolytic activity of OGA obtained through normalization on respective protein levels does not show relevant changes in the two groups. Number of animals for each condition was as follows: *n* = 6/group for western blot and RT-qPCR, *n* = 4/group for immunoprecipitation analysis, and *n* = 5/group for OGA activity assay. All bar charts reported in (B), (C), (D), (G), (H), and (I) show mean ± SEM. *p < 0.05, **p < 0.01, using Student’s *t* test

Considering the relevance of O-GlcNAcylation cycle homeostasis, many regulatory mechanisms exist to balance OGA/OGT activity, thus calibrating protein O-GlcNAc levels according to cellular status [71]. We investigated OGA/OGT functionality in the hippocampus of Ts2 mice to test whether a dysfunctional cycling could be responsible for the observed reduction of global O-GlcNAcylation. Ts2 mice showed no difference in both OGT protein expression (Fig. 4(A–B)) and transcript (Fig. 4(C)) compared to the aged-matched Eu group. Since OGT itself undergoes to O-GlcNAcylation and phosphorylation on different sites, we performed an immunoprecipitation assay to analyze OGT PTMs that could potentially affect its ability to transfer O-GlcNAc moiety [61, 66]. We noted a significant reduction in ^O-GlcNAc^OGT/OGT levels in Ts2 mice compared to the Eu group (Fig. 4(D–E); *p < 0.05, Eu vs Ts2: − 67%) and a trend on increase in p^Ser/Thr^OGT/OGT levels (Fig. 4(D–E); Eu vs Ts2: + 20%). The reduction in the O-GlcNAc/phosphorylation ratio of OGT (Fig. 4(D–E); **p < 0.01, Eu vs Ts2: − 68%) in Ts2 hippocampus suggests that the global reduction of O-GlcNAcylation might result from OGT-altered functionality. Subsequently, we analyzed the removal process of the O-GlcNAc moiety, measuring both OGA levels and enzymatic activity. OGA resulted as significantly more expressed in the hippocampus of 6-month-old Ts2 mice (Fig. 4(F–G); **p < 0.01. Eu vs Ts2: + 15%) in comparison with the control group. Furthermore, OGA protein levels also reflect the difference in mRNA transcript, which is significantly increased in Ts2 mice (Fig. 4(H); *p < 0.05; Eu Vs Ts2: + 1.20-fold change), confirming an upregulation of OGA in Ts2 mice at 6 months of age. The analysis of OGA activity demonstrated an upregulation of the global removal process of O-GlcNAc moiety (Fig. 4(I), left panel; *p < 0.01. Eu vs Ts2: + 29%) that appeared to be strictly associated with its increased expression; indeed, no changes in enzyme activity were detected after normalization on protein levels (specific activity) (Fig. 4(I), right panel). In detail, our data suggests that O-GlcNAc removal process is markedly increased in Ts2 hippocampus at 6 months of age compared to euploids as effect of increased OGA protein levels but not of OGA-specific hydrolytic activity increase. Therefore, we support that increased OGA levels promote the aberrant subtraction of O-GlcNAc moiety from serine and threonine residues, thus leading to the global reduction of O-GlcNAcylated proteins in Ts2Cje mice.

### The Aberrant O-GlcNAc/Phosphorylation Ratio of Tau and APP Drives AD-Like Neurodegeneration in Ts2Cje Mice

**Fig. 5:**
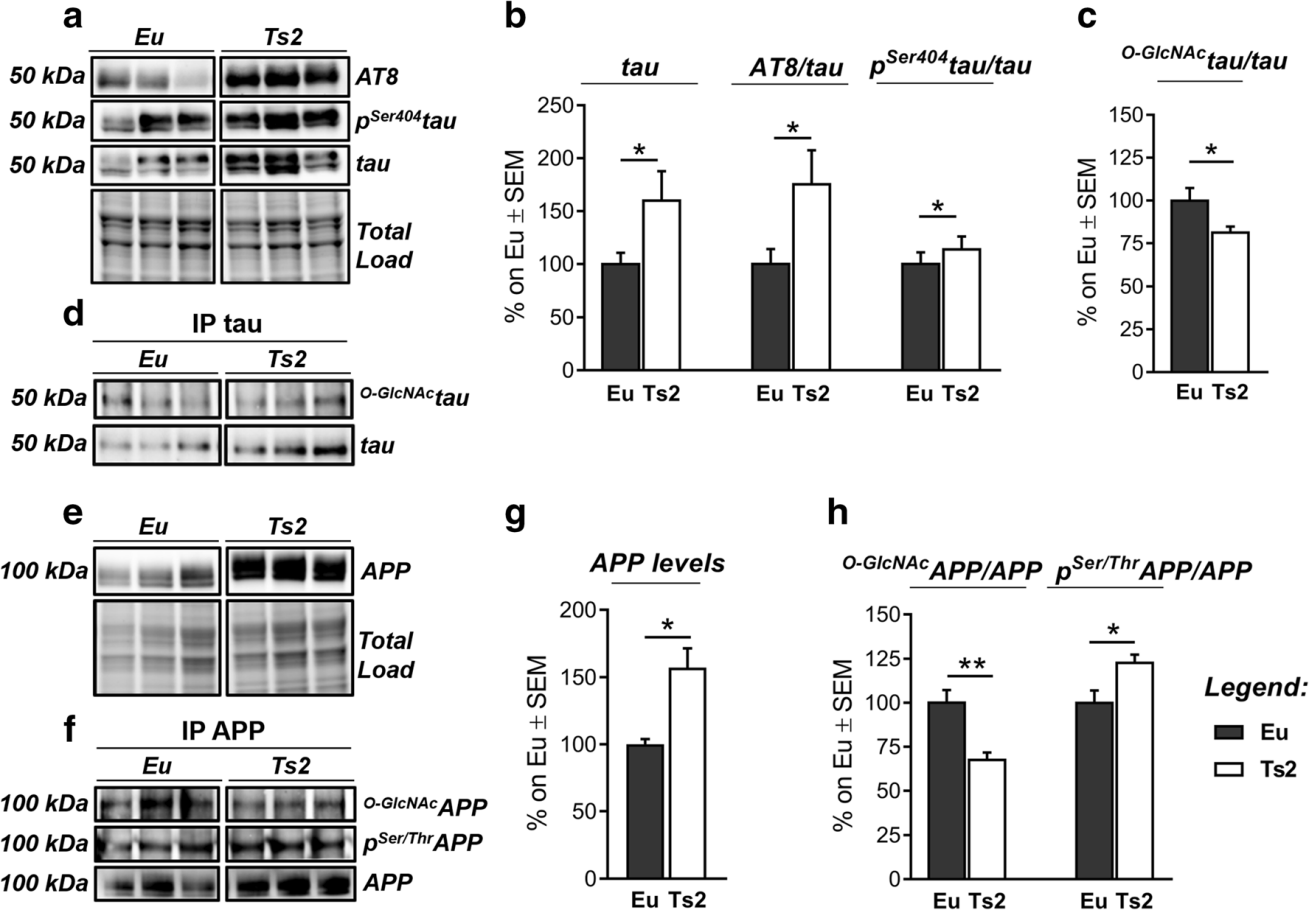
Aberrant O-GlcNAc/phosphorylation ratio of AD-related proteins in Ts2Cje mice. (A–B) Analysis of tau phosphorylated levels in Ts2 mice compared to respective euploids. Tau protein levels were significantly higher in our DS model compared to respective euploids. Moreover, increased levels of Ser202-Thr205tau/tau (AT8) and Ser404tau/tau were found in Ts2 mice in comparison to the control group. Representative blots are reported in (A). (C–D) Evaluation of ^O-GlcNAc^tau levels by immunoprecipitation analysis. A significant impairment in ^O-GlcNAc^tau/tau levels was observed in Ts2 mice compared to respective euploids. Representative blots are reported in (D). (E, G) Analysis of APP protein levels in Ts2 mice compared to respective euploids. We confirmed that APP is significantly more expressed in Ts2 mice in comparison with the control group. Representative blot is reported in (E). (F, H) Evaluation of ^O-GlcNAc^APP and p^Ser/Thr^APP levels by immunoprecipitation analysis. ^O-GlcNAc^APP/APP impairment is related with increased p^Ser/Thr^APP/APP levels in the hippocampus of Ts2 mice compared to respective euploids. Representative blots are reported in (F). Number of animals for each condition was as follows: *n* = 6/group for western blot and *n* = 4/group for immunoprecipitation analysis. All bar charts reported in (B), (C), (G), and (H) show mean ± SEM. *p < 0.05, **p < 0.01 using Student’s *t* test

The abnormal hyperphosphorylation of tau, on specific serine and threonine residues, induces protein self-assembly and gives rise to toxic NFTs, a well-established hallmark of AD-like pathology [44, 58]. Recent studies highlighted that tau phosphorylation is inversely regulated by O-GlcNAc and that tau O-GlcNAcylation plays a key role in hindering its aggregation [25, 37, 41, 57, 72]. Our data confirmed the aberrant phosphorylation of tau protein in 6-month-old Ts2 mice compared to Eu group on both Ser202-Thr205 residues (Fig. 5(A–B), AT8/tau; *p < 0.05; Eu vs Ts2: + 75%) and Ser404 (Fig. 5(A–B); *p < 0.05; Eu vs Ts2: + 14%). Furthermore, through immunoprecipitation analysis, we found that increased phosphorylation of tau is associated with a significant reduction of its O-GlcNAcylation (Fig. 5(C–D); *p < 0.05; Eu vs Ts2: − 20%). These results suggest a role for O-GlcNAc levels in the early disturbance of tau PTMs and confirm, in Ts2 neuropathology, the mutual inverse relationship between tau-reduced O-GlcNAcylated levels and its increased phosphorylation.

It was largely proven that APP undergoes O-GlcNAcylation [43] and recent advances demonstrated that increased APP O-GlcNAc levels could switch its processing from the amyloidogenic pathway to the non-amyloidogenic via, thus reducing the production of Aβ plaques [73]. Since APP is encoded on chromosome 21, the role of O-GlcNAcylation in APP processing could be further exacerbated in DS neuropathology. As previously demonstrated [74], APP protein levels were significantly higher in Ts2 mice compared to respective Eu controls (Fig. 5(E, G); **p < 0.01, Eu vs Ts2: + 60%). Subsequently, ^O-GlcNAc^APP/APP levels were demonstrated to be significantly reduced in 6-month-old Ts2 mice compared to Eu group (Fig. 5(F, H); **p < 0.01, Eu vs Ts2: − 35%) and in parallel a significant increase in p^Ser/Thr^APP/APP levels (Fig. 5(F, H); *p < 0.05, Eu vs Ts2: + 23%) was observed.

### Ts2Cje Mice Show Alteration of the HBP and the Induction of the Insulin Cascade

**Fig. 6:**
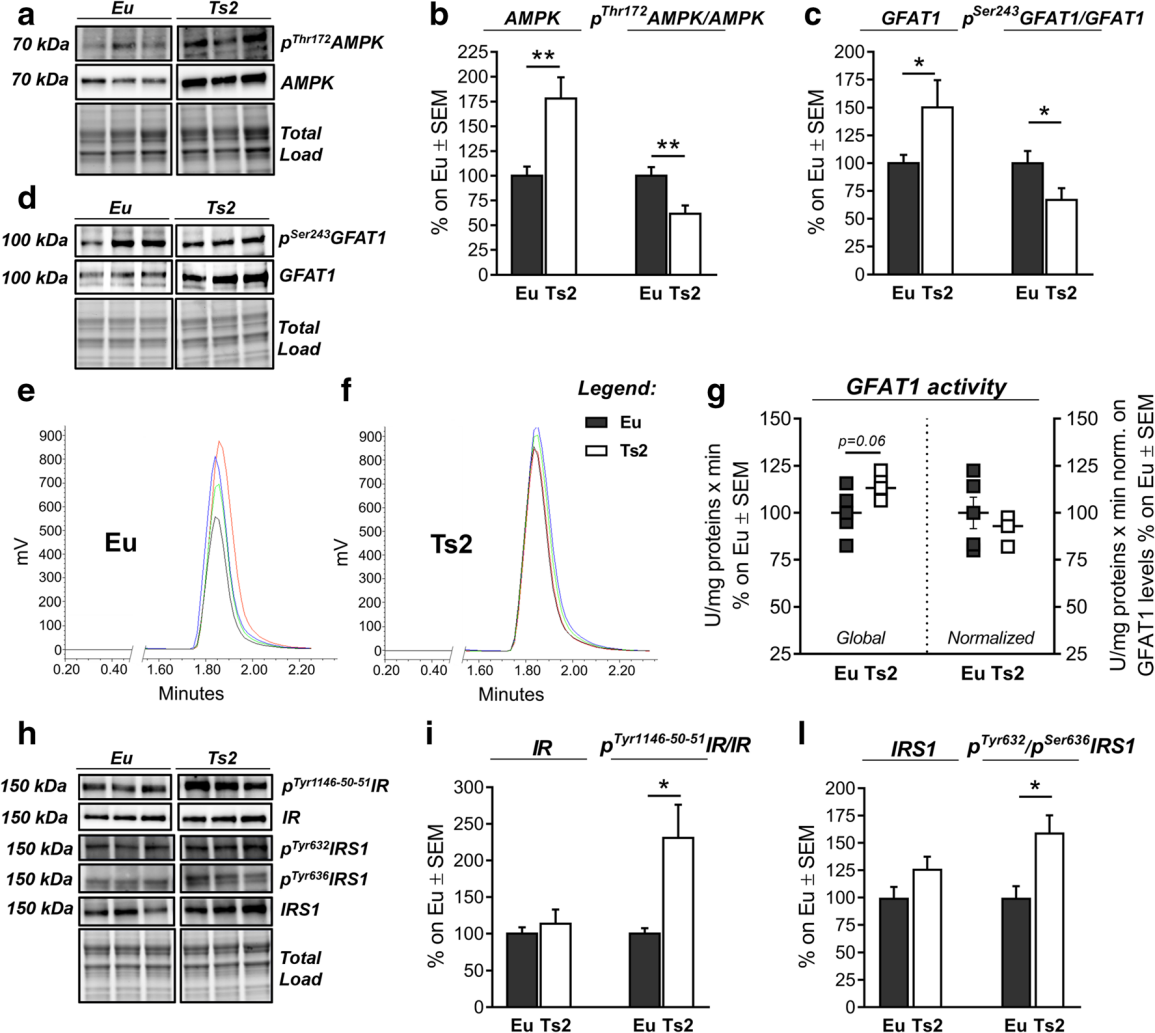
HBP flux is impaired in Ts2Cje mice together with a hyperactivation of the insulin cascade. (A–B) Analysis of AMPK activation status in Ts2 mice compared to respective euploids. A significant increase in the AMPK protein levels was observed in Ts2 mice compared to Eu, together with a significant impairment in p^Thr172^AMPK/AMPK levels, thus resulting in reduced AMPK activation. Representative blots are reported in (A). (C–D) Analysis of GFAT1 activation status in Ts2 mice compared to respective euploids. A significant increase in GFAT1 levels was observed in Ts2 mice compared to the control group, together with an impairment of p^Ser243^GFAT1/GFAT1 ratio, resulting in reduced GFAT1 inhibition. Representative blots are reported in (D). (E–G) GFAT1 activity assay. GFAT1 global activity showed a trend of increase in Ts2 hippocampus compared to respective euploids, while GFAT1 enzymatic normalized on corresponding protein expression levels showed no alteration. Representative spectra of GFAT1-synthetized glucosamine-6-phosphate for both Ts2 and euploid animals are reported in (E) and (F) and respective bar graph of global and normalized activity (G). (H–L) Analysis of the insulin cascade in Ts2 mice compared to respective euploids. A significant increase in the phosphorylated levels of insulin receptor (Tyr1146-1150-1151)/IR was observed in Ts2 mice compared to Eu (I). Ts2 mice also showed an increase in the activation of the insulin receptor substrate (IRS-1), with increased ratio between phosphorylated levels on activatory site (Tyr632) and inhibitory site (Ser636) (L). Representative blots are reported in (H). Number of animals for each condition were as follow: *n* = 6/group for both western blot analysis and *n* = 5/group for GFAT1 activity assay. All bar charts reported in (B), (C), (G), (I), and (L) show mean ± SEM. *p < 0.05, **p < 0.01, using Student’s *t* test

Since DS is characterized by a significant altered metabolic profile, with a prevalence of less efficient fermentative metabolism [75, 76], we decided to analyze the activation state of AMP-activated protein kinase (AMPK) in our Ts2 model and its relevance in the control of HBP at early stage of disease. A significant increase in AMPK protein levels was observed in the hippocampus of 6-month-old Ts2 compared to the Eu group (Fig. 6(A–B); **p < 0.01, Eu vs Ts2: + 78%), together with a significant reduction of AMPK activation measured by its p^Thr172^AMPK levels normalized on respective protein levels (Fig. 6(A–B); **p < 0.01, Eu vs Ts2: + 40%). The analysis of GFAT1 protein levels showed a significant difference among the two groups (Fig. 6(C–D); *p < 0.05, Eu vs Ts2: + 50%), while GFAT1 phosphorylation on Ser 243, which is controlled by AMPK and regulates the inhibition of its catalytic activity, showed a significant reduction in Ts2 mice compared to euploids (Fig. 6(C–D); *p < 0.05; Eu Vs Ts2: + 34%). In order to evaluate GFAT1 activation state, we took advantage of an HPLC-based method to measure its direct enzymatic product. In accordance with the lack of AMPK inhibitory effect, a trend of increase was observed in GFAT1 global ability to synthetize glucosamine-6-phosphate in Ts2 mice compared to the Eu group (Fig. 6(E–G)). However, since GFAT1 protein levels change between Ts2 and Eu animals, the activity was also normalized on respective protein levels demonstrating no significant alterations. To fully clarify if Ts2 shows metabolic alterations that can impact the HBP and the O-GlcNAcylation process, we evaluated the insulin cascade and/or glucose uptake. At first, we analyzed in 6-month-old animals the phosphorylated levels of IR on Tyr1146-1150-1151 to assess its activation state: a significant increase in the phosphorylated levels of IR/IR was observed in Ts2 hippocampus compared to respective euploids (Fig. 6(H–I); *p < 0.05, Eu vs Ts2: + 130%), while no significant changes were noticed on IR protein levels in the two groups. The evaluation of IR’s direct substrate, IRS-1, demonstrated a significant increase in p^Tyr632^IRS-1 (activatory site) compared to p^Ser636^IRS (inhibitory site) in Ts2 mice compared to the Eu group (Fig. 6(H, L); *p < 0.05, Eu vs Ts2: + 60%). No relevant differences were observed in IRS-1 protein levels between the two groups. According to our results, Ts2 mice at 6 months of age suggest the increased activation of the insulin cascade compared to respective euploids. The subsequent analysis of GSK3β demonstrated the inhibition of the protein (Sup. Fig. 5) supporting, as reported in human brain, an uncoupling of the signal [22]. Furthermore, the observed activation of the insulin cascade in Ts2 mice was associated with unaltered levels of brain glucose transporters and GLUT4 translocation to the membrane as suggested by unaltered p^Thr142^As160/AS160 ratio in Ts2 mice hippocampus (Sup. Fig. 6).

### Intranasal Thiamet G Rescued Aberrant Protein O-GlcNAcylation and OGA Activity in 6-Month-Old Ts2Cje Mice

**Fig. 7:**
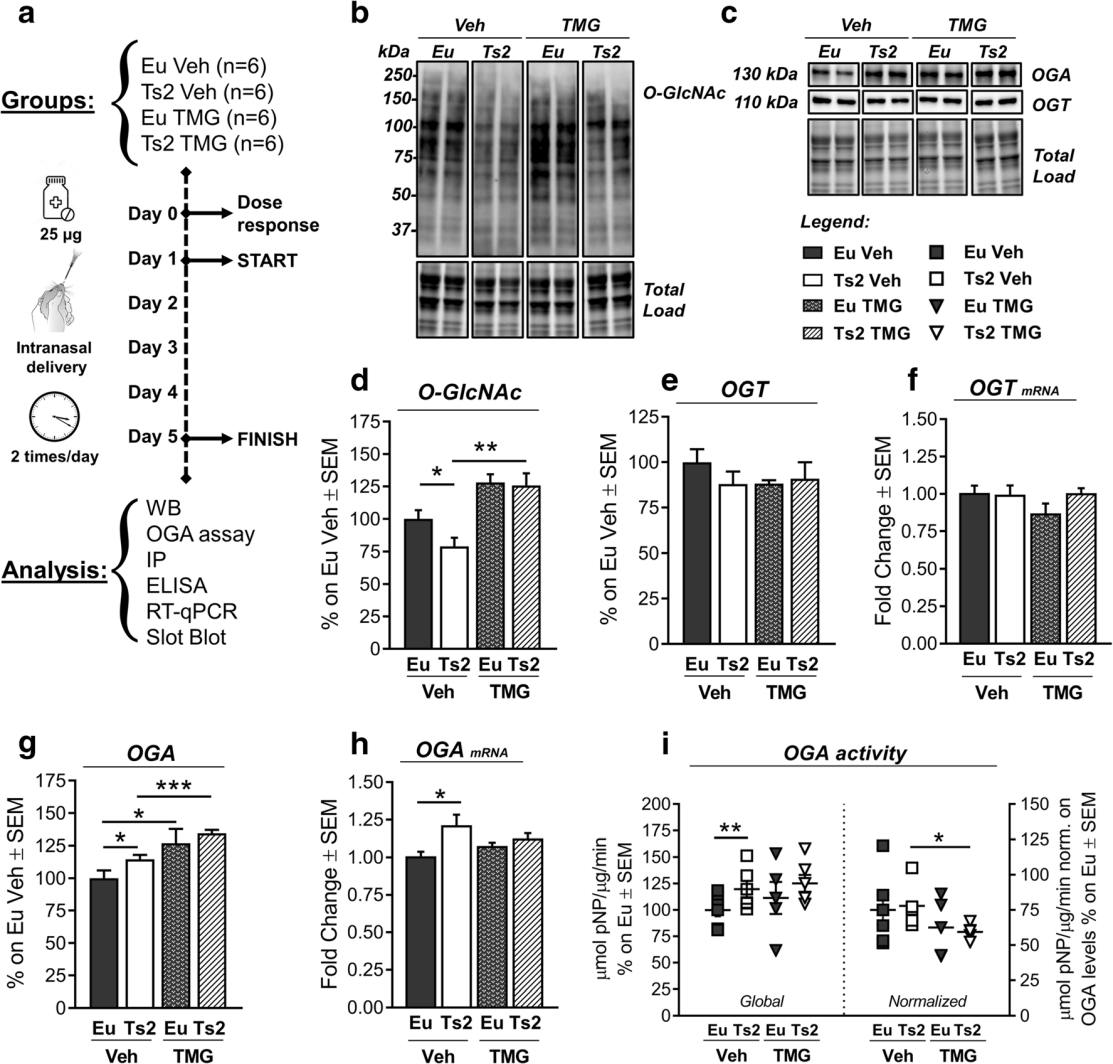
Short-term TMG intranasal treatment rescued protein O-GlcNAcylation and OGA activity in 6-month-old Ts2Cje mice. (A) Schematic representation of the short-term TMG intranasal treatment. After a single dose-response study to assess the correct TMG dose (Sup Fig. 7), 6-month-old animals were treated twice a day with vehicle solution (Veh; PBS 1X solution) or TMG (25 μg Thiamet G solution) for 5 days. Animals were divided according to their genotype and intranasal treatment received in the following groups: Eu Veh, Ts2 Veh, Eu TMG, Ts2 TMG. Samples were then collected for subsequent analysis. (B, D) Analysis of protein O-GlcNAcylation levels after TMG treatment. TMG intranasal treatment rescued protein O-GlcNAcylation in Ts2 TMG compared to Ts2 Veh. An increase of protein O-GlcNAcylation levels was also observed in Eu treated with TMG in comparison to Eu Veh. Representative blot is reported in (B). (C) Representative blots of OGA and OGT protein levels after TMG treatment. (E–F) Analysis of OGT protein levels and transcript after TMG treatment. No changes in OGT protein levels (E) and transcript (F) were observed in treated mice. Representative blot is reported in (C). (G–H) Analysis of OGA protein levels and transcript after treatment. TMG-treated triggered a significant increase in OGA protein levels both in Eu and Ts2 mice (G), while no changes were observed in OGA transcript levels, following TMG administration (H). Representative blot is reported in (C). (I) Analysis of OGA activity after treatment. TMG induced a significant reduction of OGA enzyme-specific activity in TMG-treated Ts2 mice compared to Ts2 mice treated with vehicle. OGA enzyme-specific activity was obtained for each group through normalization on corresponding protein expression levels. Number of animals for each condition was as follows: *n* = 6/group for western blot analysis, *n* = 5/group for RT-qPCR and OGA activity assay. All bar charts reported in (D), (E), (F), (G), (H), and (I) show mean ± SEM. *p < 0.05, **p < 0.01, ***p < 0.001 using Student’s *t* test

Our results demonstrated that aberrant protein O-GlcNAcylation occurs in Ts2 mice as a result of increased OGA expression, which plays a significant role in the onset of AD-related markers. Hence, to question the possible neuroprotective effects of rescuing protein O-GlcNAcylation in Ts2 neuropathology, we performed a short-term intranasal treatment with Thiamet G (TMG), a potent and selective OGA inhibitor. Both 6-month-old Ts2 and respective Eu were administered by intranasal route with vehicle solution (Veh) or 25 μg of TMG (Fig. 7(A)), with the aim of targeting the brain directly and avoiding effects on peripheral organs [77] that might behave differently in terms of O-GlcNAcylation. After 5 days of treatment, we observed that TMG treatment was able to rescue lower levels of O-GlcNAcylated proteins in Ts2 hippocampus, restoring euploid-physiological levels (Fig. 7(B, D); **p < 0.01, Ts2 Veh vs Ts2 TMG: + 46%), affecting neither OGT protein levels (Fig. 7(C, E)) nor transcript (Fig. 7(F)). Accordingly, 2-way ANOVA analysis supported the effect of TMG treatment in rising O-GlcNAcylated protein levels (Table 2; *F* (1,21) = 21.25, ***p < 0.001). In agreement with our hypothesis, the analysis of OGA enzymatic activity demonstrated the efficacy of the intranasal TMG administration in inhibiting the aberrant removal of O-GlcNAc moiety from serine and threonine residues of hippocampal proteins. Indeed, TMG treatment induced a significant reduction of OGA activity normalized on protein levels (specific activity) in Ts2-treated mice compared to respective Ts2 treated with vehicle solution (Fig. 7(I), right panel; *p < 0.05: Ts2 Veh vs Ts2 TMG: − 24%). In any event, due to increased OGA expression levels in treated-Ts2Cje mice, we did not observe reduction on global OGA enzyme activity. Furthermore, we confirmed a genotype-dependent upregulation of both OGA protein levels (Fig. 7(C, G)) and transcript (Fig. 7(H)) in Ts2 mice compared to euploids treated with vehicle (Table 2; OGA protein: *F* (1,22) = 3.7, *p < 0.05; OGA mRNA: *F* (1,20) = 5.3, *p < 0.05). Intriguingly, as described elsewhere [78, 79], TMG treatment was able to trigger a significant increase of OGA protein levels in both Eu (Fig. 7(C, G); *p < 0.05, Eu Veh vs Eu TMG: + 27%) and Ts2 animals (Fig. 7(C, G); ***p < 0.001, Ts2 Veh vs Ts2 TMG: + 20%). Furthermore, an effect of TMG treatment in increasing OGA protein levels was also assessed by 2-way ANOVA analysis (Table 2; *F* (1,22) = 16.8, ***p < 0.001). Surprisingly, upregulated OGA protein levels did not significantly reflect OGA mRNA levels both in Eu and Ts2 mice treated with TMG (Fig. 7(H)). The effect of TMG on OGA supports the occurrence of a TMG-induced compensatory mechanisms as a result of the inhibition of OGA catalytic activity [80].

**Table 2:** 2-way ANOVA analysis in Eu and Ts2 mice treated with Veh and TMG

2-way ANOVA analysis			
Target of interest	Genotype (Eu vs Ts2)	Treatment (Veh vs TMG)	Interaction
O-GlcNAc	*F* (1,21) = 2.055 *p* = 0.1665	F (1,21) = 21.25 ***p = 0.0002	*F* (1,21) = 1.327 *p* = 0.2622
OGA activity/OGA	*F* (1,17) = 0.0001521 *p* = 0.9903	*F* (1,17) = 3.468 *p* = 0.0799	*F* (1,17) = 0.1305 *p* = 0.7224
OGT	*F* (1,19) = 0.3515 *p* = 0.5602	*F* (1,19) = 0.3289 *p* = 0.5730	*F* (1,19) = 0.8781 *p* = 0.3605
OGA	*F (1*,22) = 3.710 *p = 0.0498	*F (1*,22) = 16.83 ***p = 0.0005	*F* (1,22) = 0.4029 *p* = 0.5321
OGT *mRNA*	*F* (1,20) = 0.9879 *p* = 0.3321	*F* (1,20) = 1.047 *p* = 0.3184	*F* (1,20) = 1.427 *p* = 0.2462
OGA *mRNA*	*F (1*,20) = 5.337 *p = 0.0317	*F* (1,20) = 0.02546 *p* = 0.8748	*F* (1,20) = 1.985 *p* = 0.1742
AT8/tau	*F (1*,19) = 4.441 *p = 0.0486	*F* (1,19) = 0.08656 *p* = 0.7718	*F* (1,19) = 1.069 *p* = 0.3141
p^Ser404^/tau	*F* (1,21) = 0.1369 *p* = 0.7150	*F* (1,21) = 1.131 *p* = 0.2997	*F (1*,21) = 7.610 *p = 0.0118
β-CTF/α-CTF	*F (1*,17) = 24.06 ***p = 0.0001	*F (1*,17) = 9.043 **p = 0.0079	*F* (1,17) = 0.001069 *p* = 0.9743
Aβ 42	*F* (1,20) = 0.6361 *p* = 0.4345	*F (1*,20) = 8.009 *p = 0.0103	*F* (1,20) = 0.01148 *p* = 9157
PSD95	*F* (1,18) = 0.08181 *p* = 0.7781	*F (1*,18) = 7.727 *p = 0.0124	*F* (1,18) = 0.0691 *p* = 0.7956
Syntaxin 1A	*F* (1,18) = 2.324 *p* = 0.1448	*F (1*,18) = 16.26 ***p = 0.0008	*F* (1,18) = 1.398 *p* = 0.2524
BDNF	*F* (1,19) = 1.115 *p* = 0.3043	*F (1*,19) = 14.05 **p = 0.0014	*F (1*,19) = 23.08 ***p = 0.0001
Atg7	*F* (1,21) = 0.05321 *p* = 0.8198	*F (1*,21) = 14.48 **p = 0.0010	*F* (1,21) = 0.1.574 *p* = 0.2234
Beclin-1	*F* (1,23) = 0.005176 *p* = 0.9433	*F (1*,23) = 16.48 ***p = 0.0005	*F* (1,23) = 2.745 *p* = 0.1111
LC3-II/I	*F (1*,21) = 7.926 *p = 0.0104	*F (1*,21) = 5.861 *p = 0.0246	*F (1*,21) = 13.85 **p = 0.0013
SQSTM1	*F* (1,23) = 2.521 *p* = 0.1260	*F* (1,23) = 0.9074 *p* = 0.3507	*F (1*,23) = 6.288 *p = 0.0197
3-NT	*F* (1,21) = 2.401 *p* = 0.1362	*F* (1,21) =1.65 *p* = 0.2130	*F (1*,21) = 5.68 *p = 0.0180
HNE adducts	*F (1*,22) = 18.75 ***p = 0.0003	*F* (1,22) = 2.289 *p* = 0.1445	*F* (1,22) = 0.09204 *p* = 0.7644

Significant *p*-values are reported in italics

### Intranasal Thiamet G Rescued Aberrant APP and Tau PTMs in Ts2Cje Hippocampus

**Fig. 8:**
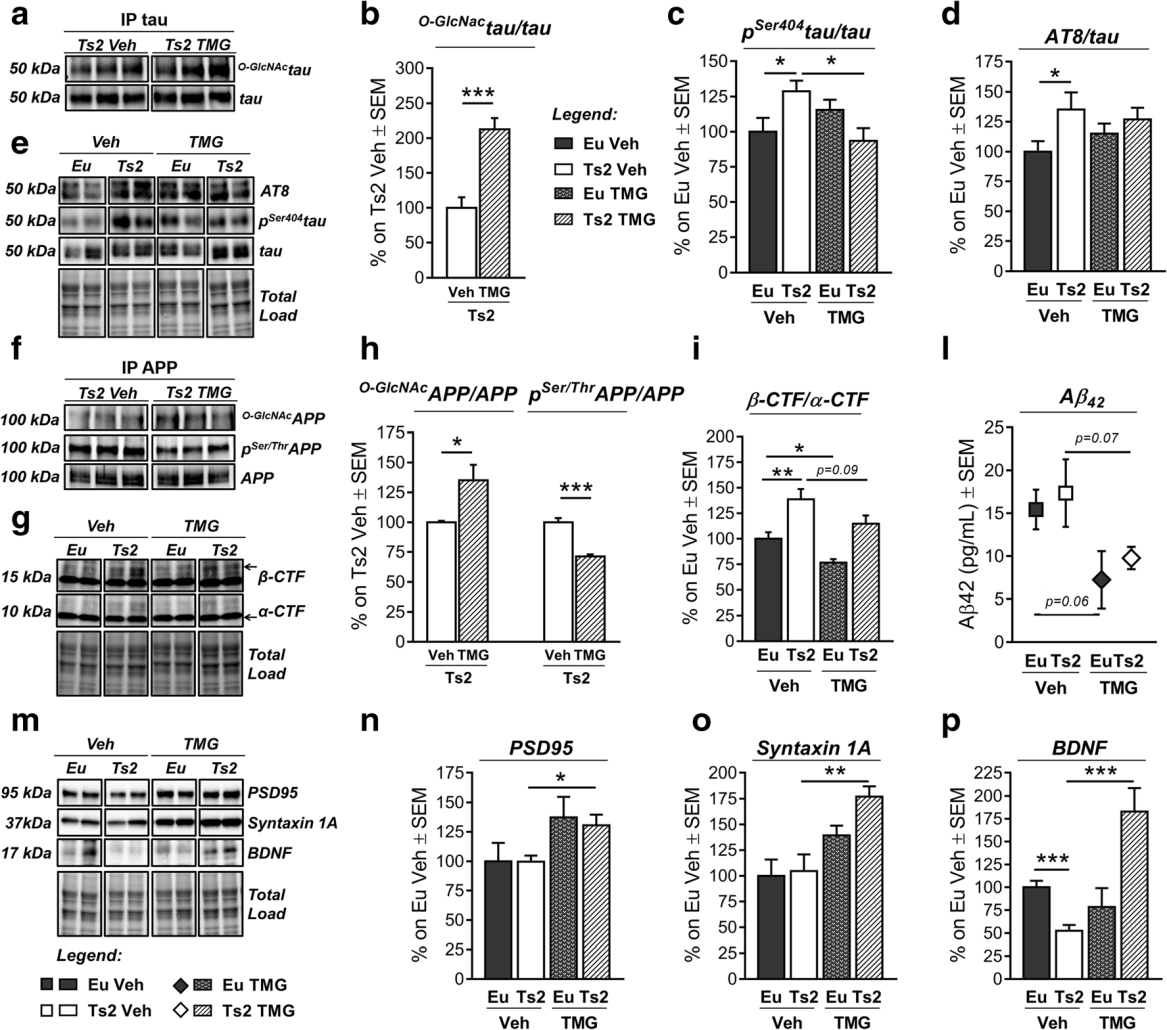
Short-term TMG intranasal treatment rescued aberrant tau and APP PTMs and increased synaptic proteins expression in Ts2Cje mice. (A–B) Evaluation of ^O-GlcNAc^tau levels by immunoprecipitation analysis after TMG treatment. TMG intranasal treatment induced a significant increase in ^O-GlcNAc^tau/tau levels of TMG-treated Ts2 compared to vehicle-administered Ts2 mice. Representative blots are reported in (A). (C–E) Analysis of tau phosphorylated levels after TMG treatment. No changes were observed in tau phosphorylation levels on Ser-202-Thr208tau/tau of TMG-treated Ts2 (C), while a significant reduction was reported in Ser404tau/tau of Ts2 TMG compared to Ts2 Veh (D). Representative blots are reported in (E). (F, H) Evaluation of ^O-GlcNAc^APP and p^Ser/Thr^APP levels after TMG treatment. A significant increase in ^O-GlcNAc^APP/APP levels was measured, together with a significant reduction of p^Ser/Thr^APP/APP levels in Ts2 TMG compared to Ts2 Veh. Representative blots are reported in (F). (G, L) Evaluation of APP cleavage trough the measure of β-CTF/α-CTF ratio and soluble Aβ 1-42 peptide after TMG treatment. A significant increase in β-CTF/α-CTF ratio was observed in Ts2 Veh compared to Eu Veh, confirming the preferential amyloidogenic processing of APP in our DS model. The increased ^O-GlcNAc^APP/APP levels reflect the reduction of β-CTF/α-CTF ratio in treated Ts2 mice, confirming the ability of TMG treatment to favor the non-amyloidogenic cleavage of APP. Furthermore, evaluation of soluble Aβ 1-42 peptide by ELISA showed a trend of reduction in both Euploid and Ts2 mice treated with TMG in comparison to the respective group administered with Veh, confirming the effect of intranasal TMG treatment in modulating APP’s fate. Representative blots are reported in (G). (M–P) Evaluation of PDS95, Syntaxin 1A, and BDNF protein levels after TMG treatment. A significant increase in both PSD95 and Syntaxin 1A protein levels was observed in Ts2 mice treated with TMG compared to Ts2 animals treated with Veh. Interestingly, TMG treatment was also able to rescue the impairment of BDNF protein levels that was observed in Ts2 Veh compared to Eu Veh. Indeed, TMG-treated Ts2 mice showed a significant increase in BDNF protein levels in comparison to the Ts2 Veh group, confirming an effect of TMG treatment in inducing synaptic-related proteins and neurotrophic factors. Representative blots are reported in (M). Number of animals for each condition was as follows: *n* = 6/group for western blot and ELISA analysis, *n* = 4/group for immunoprecipitation analysis. All bar charts reported in (B), (C), (D), (H), (I), (L), (N), (O), and (P) show mean ± SEM. *p < 0.05, **p < 0.01, ***p < 0.001 using Student’s *t* test

Subsequently, we evaluated the consequence of TMG treatment on tau. We observed a considerable increase of ^O-GlcNAc^tau/tau levels in TMG-treated Ts2 mice compared to Ts2 administered with vehicle solution (Fig. 8(A–B); ***p < 0.001, Ts2 Veh vs Ts2 TMG: + 113%). Since pharmacological elevation of ^O-GlcNAc^tau through OGA inhibitors was related with reduced toxic forms of tau [36], we then measured the levels of phosphorylated tau. A significant reduction of the levels of tau phosphorylated on Ser404 was detected in Ts2 TMG-treated mice compared to respective trisomic animals treated with vehicle (Fig. 8(C, E); *p < 0.05, Ts2 Veh vs Ts2 TMG: − 35%) with a mutual interaction between genotype and treatment described by 2-way ANOVA analysis (Table 2; *F* (1,21) = 7.6, *p < 0.05). On the contrary, no changes in Ser202-Thr205 phosphorylation were observed in TMG-treated Ts2 compared withTs2 Veh (Fig. 8(D–E): AT8/tau). As regards APP, we observed that the short-term intranasal treatment with TMG induced a significant increase in the levels of ^O-GlcNAc^APP/APP in Ts2 mice compared to the corresponding vehicle group (Fig. 8(F, H); *p < 0.05, Ts2 Veh vs Ts2 TMG: + 35%), which was in line with a significant reciprocal inverse reduction of p^Ser/Thr^APP/APP levels (Fig. 8(F, H); ***p < 0.001, Ts2 Veh vs Ts2 TMG: − 30%). Since site-specific phosphorylation on serine and threonine residues are known to influence APP’s fate [81–83], we presumed that TMG intranasal treatment could exert beneficial effects on APP cleavage on Ts2Cje, as reported for AD murine models [84, 85]. To support our hypothesis, we analyzed APP cleavage products measuring both α-CTF and β-CTF. As expected, Ts2 Veh mice showed a significant increase in β-CTF/α-CTF ratio compared to respective euploids (Fig. 8(G, I); **p < 0.01, Eu Veh Vs Ts2 Veh: + 40%) with a relevant contribution of genotype (Table 2; *F* (1,17) = 24.1, ***p < 0.001) that confirms a tendency toward the APP amyloidogenic processing in our DS model. According to our result, TMG intranasal administration induced a significant reduction of β-CTF/α-CTF ratio in the euploid group (Fig. 8(G, I); *p < 0.05, Eu Veh vs Eu TMG: − 24%) but only a trend of decrease in Ts2-treated mice (Fig. 8(G, I)). However, an effect of TMG treatment in reducing β-CTF/α-CTF ratio was confirmed by 2-way ANOVA analysis (Table 2; *F* (1,17) = 9.1, **p < 0.01). Our data suggest that the reduction of β-CTF/α-CTF ratio in the treated groups is mainly due to an increase of the α-CTF fragment rather than a significant reduction in β-CTF levels (Sup. Fig 8), indicating that the increase in ^O-GlcNAc^APP levels may favor the non-amyloidogenic cleavage of the protein. Further, to account for the ability of TMG on reducing Aβ formation, we analyzed soluble Aβ 1-42 after intranasal treatment. To note, previous studies on DS models demonstrated that mice do not exhibit Aβ plaques within the brain, while the increase of soluble Aβ might occur after mice reach middle age [86, 87]. Our analysis demonstrates a slight but not significant increase of Aβ 1-42 in Ts2 mice compared to respective euploids (Fig. 8(L); Eu Veh vs Ts2 Veh: + 1.9 pg/mL), while the treatment with TMG showed a trend of decrease in both Eu (Fig. 8(L); *p* = 0.06, Eu Veh vs Eu TMG: − 8.2 pg/mL) and Ts2 animals (Fig. 8(L); *p* = 0.07, Ts2 Veh vs Ts2 TMG: − 7.6 pg/mL) suggesting the potential efficacy of the compound. In line with this trend, an effect of TMG treatment on Aβ 1-42 peptide was also confirmed by 2-way ANOVA analysis (Table 2; *F* (1,20) = 8.0, *p < 0.05).

### Thiamet G Increases the Levels of PSD95, Syntaxin 1A, and BDNF in Ts2Cje Mice

In order to further characterize the possible neuroprotective effects of our treatment, we also analyzed the expression of synapse-related proteins in TMG-treated mice. In detail, TMG treatment proved to significantly raise PSD95 protein expression levels in the hippocampus of Ts2 TMG mice (Fig. 8(M–N); *p < 0.05, Ts2 Veh vs Ts2 TMG: + 31%) and induce the same significant increase of Syntaxin 1A protein levels in TMG-treated mice compared to the Ts2 Veh group (Fig. 8(M, O); **p < 0.01, Ts2 Veh vs Ts2 TMG: + 72%). Consistent with the above data, 2-way ANOVA analysis confirmed an effect of TMG treatment for both PSD95 and Syntaxin 1A (Table 2; PSD95: *F* (1,18) = 7.7, *p < 0.05; Syntaxin 1A: *F* (1,18) = 16.3, ***p < 0.001). Interestingly, previous studies on the Ts65Dn model correlated reduced brain-derived neurotrophic factor (BDNF) with poor spatial memory in 6-month-old animals [88] and BDNF-mimetic therapy proved to rescue synaptic plasticity and memory deficits in Ts65Dn mice [89]. In this scenario, our analysis on 6-month-old Ts2 animals confirmed a significant reduction of BDNF protein levels in the hippocampal region of trisomic mice treated with Veh compared to equally treated Eu (Fig. 8(M, P); ***p < 0.001, Eu Veh vs Ts2 Veh: − 37%). Furthermore, TMG-treated Ts2 mice showed significant higher levels of BDNF protein in comparison to respective Ts2 treated with vehicle (Fig. 8(M, P); ***p < 0.001, Ts2 Veh vs Ts2 TMG: + 83%). This effect of TMG treatment was also demonstrated by 2-way ANOVA analysis (Table 2; *F* (1,19) = 14, **p < 0.01), and also a synergic effect of both treatment and genotype was observed (Table 2; *F* (1,19) = 23, ***p < 0.001). Overall, these data suggest that TMG treatment could exert its benefits also through the induction of synapse-related proteins, possibly recovery Ts2 cognitive deficits.

### Thiamet G Treatment Boosts Autophagic Clearance and Reduces Oxidative Damage in 6-Month-Old Ts2Cje Mice

**Fig. 9:**
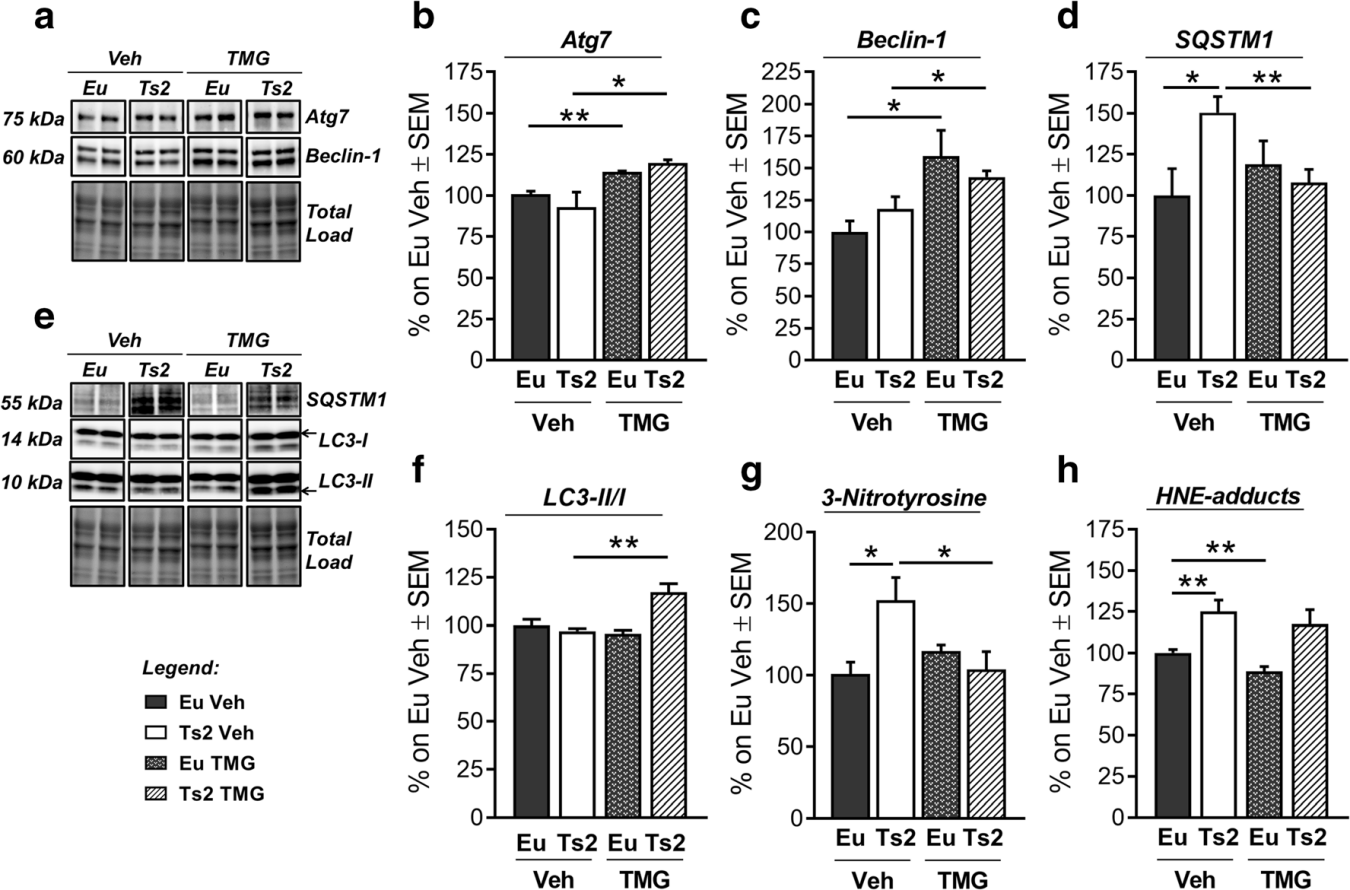
Short-term TMG intranasal treatment boosted autophagic clearance and reduced oxidative damage in Ts2Cje mice. (A–C) Analysis of the initial steps of autophagy machinery after TMG treatment. TMG intranasal treatment proved to induce initial steps of autophagic flux by significantly increasing Atg7 (B) and Beclin-1 (C) protein levels in TMG-treated mice compared to animals treated with vehicle solution, independently form the genotype. Representative blots are reported in (A). (D–F) Analysis of autophagic flux efficiency after TMG treatment. Six-month-old Ts2 mice showed an accumulation of SQSTM1 protein levels compared to respective euploid treated with vehicle, suggesting a failure in autolysosomal degradation. TMG showed to significantly reduce SQSTM1 levels, reactivating the autophagic flux in Ts2 mice (D). Furthermore, our treatment significantly increased LC3II/I ratio in TMG-treated Ts2 compared to Ts2 administered with Veh, confirming an amelioration of autophagosome maturation (F). Representative blots are reported in (E). (G) Analysis of 3-nitrotyrosine levels after TMG treatment. Intriguingly, high levels of oxidatively modified proteins are decreased by the administration of TMG in Ts2 mice which proved to reduce 3-NT levels in comparison to Ts2 administered with vehicle solution. Global 3-NT levels are measured by slot blot technique. (H) Analysis of HNE adduct levels after TMG treatment. High levels of HNE adducts characterized Ts2 animals compared to respective Eu Veh. TMG treatment proved to significantly reduce HNE adducts in euploid animals together with a trend of reduction in the TMG-treated Ts2 group. Global HNE adducts are measured by slot blot technique. Number of animals for each condition was as follows: *n* = 6/group for western blot analysis and slot blot analysis. All bar charts reported in (B), (C), (D), (F), (G), and (H) show mean ± SEM. *p < 0.05, **p < 0.01 using Student’s *t* test

Furthermore, we evaluated the influence of O-GlcNAc rescue on autophagy induction and protein oxidative damage. Taking into account recent findings by Zhu et al. regarding the ability of TMG to boost autophagy [90], we evaluated possible implications of our intranasal TMG treatment in Ts2 mice. We observed a relevant increase in Atg7 protein levels both in Eu mice treated with TMG (Fig. 9(A–B); **p < 0.01, Eu Veh vs Eu TMG: + 14%) and Ts2 equally treated (Fig. 9(A–B); *p < 0.05, Ts2 Veh vs Ts2 TMG: + 27%). In line with the promotion of autophagic initial steps, TMG treatment also stimulated a significant increase in Beclin-1 protein levels both in euploids mice (Fig. 9(A, D); *p < 0.05, Eu Veh vs Eu TMG: + 60%) and respective Ts2 animals (Fig. 9(A, D); *p < 0.05, Ts2 Veh vs Ts2 TMG: + 25%). Consistent with the above data, 2-way ANOVA confirmed that Atg7 and Beclin-1 are increased as an effect of the treatment (Table 2; Atg7: *F* (1,22) = 14.5, ***p < 0.001; Beclin-1: *F* (1,22) = 16.5 ***p < 0.001). Subsequently, we analyzed changes in LC3 protein by measuring its cleveated forms as index of autophagosome maturation [91]. Interestingly, TMG treatment proved to significantly increase LC3II/I ratio in Ts2 mice (Fig. 9(E–F); **p < 0.01, Ts2 Veh vs Ts2 TMG: + 20%) as a result of treatment administration (Table 2; *F* (1,21) = 5.7, *p < 0.05) together with a synergistic effect of treatment and genotype (Table 2; *F* (1,21) = 13.9, **p < 0.01). Afterwards, we evaluated the possible effects of TMG treatment in autolysosomal degradation efficacy, measuring SQSTM1 levels. We observed a relevant accumulation of SQSTM1 protein in Ts2 mice compared to age-matched controls treated with Veh solution (Fig. 9(D–E); *p < 0.05, Eu Veh vs Ts2 Veh: + 50%), suggesting a failure in autolysosomal clearance in our DS model. Intriguingly, TMG treatment proved to boost autophagic flux by significantly reducing SQSTM1 levels in TMG-treated Ts2 mice (Fig. 9(D–E); **p < 0.01, Ts2 Veh vs Ts2 TMG: − 43%), reactivating autolysosomal degradation. Moreover, 2-way ANOVA analysis revealed a combined effect of both genotype and treatment in the observed changes of SQSTM1 protein levels (Table 2; *F* (1,23) = 6.3, *p < 0.05). Among all the different characteristics of DS neuropathology, an increase of protein oxidative damage was observed [92–95]. Since autophagy induction is considered one of the strategies able to promote the degradation of toxic aggregates [96], we evaluated the benefits of our TMG treatment in reducing oxidatively modified proteins which is Ts2 mice. As expected, 6-month-old Ts2 mice showed high levels of 3-nitrotyrosine (3-NT) compared to age-matched controls (Fig. 9(G); *p < 0.05, Eu Veh vs Ts2 Veh: + 51%). TMG treatment showed ability to reduce 3-NT levels in the hippocampus of Ts2-treated mice (Fig. 9(G); *p < 0.05, Ts2 Veh vs Ts2 TMG: − 48%). Moreover, 2-way ANOVA analysis pointed out a combined effect of both genotype and treatment on 3-NT levels (Table 2; *F* (1;21) = 5.7, *p < 0.05). In line with this result, HNE adduct levels were also higher in Ts2 mice compared to respective euploids (Fig. 9(H); *p < 0.05, Eu Veh vs Ts2 Veh: + 25%), with a remarkable effect of the genotype (Table 2; *F* (1,22) = 18.8, ***p < 0.0001). However, TMG treatment was not able to rescue HNE adduct levels in Ts2 animals. Our data suggest a possible implication of TMG-induced autophagy in the clearance of nitrated proteins.

## Discussion

In the last decade, a great deal of effort has been made to investigate the role of protein O-GlcNAcylation in neurodegenerative diseases; however, no evidence is available on its possible implications in DS neuropathology. Our analysis in Ts2Cje mice showed a persistent reduction of total GlcNAc bound to protein in each area of the hippocampal region, suggesting the alteration of O-GlcNAcylation homeostasis as an early molecular event that could increase susceptibility to neurodegenerative phenotypes. Previous reports demonstrated that decreased protein O-GlcNAcylation in the hippocampal region drives synaptic and cognitive decline in the aging brain, facilitating later onset of dementia [97]. Among the proposed mechanisms through which loss of O-GlcNAcylation promotes neurodegenerative processes, the extensive interplay of this PTM with protein phosphorylation has special relevance [38, 45, 58]. In agreement, our analysis showed increased global protein phosphorylation on Ser/Thr residues and the imbalance of the O-GlcNAcylation/phosphorylation equilibrium in the hippocampal area, recapitulating the alterations observed in the brain of both AD humans and murine models [23, 26, 28, 38, 41, 55, 98]. Most interesting, the reciprocal interplay between protein O-GlcNAcylation and phosphorylation is not only related to the competitive modification of the same residues but also to the ability of each modification to regulate the other’s enzymatic machinery. Recently, insulin stimulation of 3T3-L1 adipocytes was proved to increase both OGT tyrosine phosphorylation and catalytic activity [99]. Furthermore, Kaasik et al. found that GSK3β can enhance OGT activity by phosphorylation on Ser residues [61]. However, the decreased O-GlcNAcylation of OGT, observed in our model, may alter the sites available for phosphorylation independently of GSK3β, thus affecting its activity. Among control mechanisms of O-GlcNAc cycling, fluctuations in protein O-GlcNAcylation are known to affect both OGT and OGA transcription in physiological context, in the direction of compensating the imbalance [71, 80]. Recent evidence has demonstrated that O-GlcNAc perturbation affects the splicing of the highly conserved detained introns in OGT and OGA in order to control mRNA abundance, altering OGT and OGA protein levels to buffer changes in O-GlcNAc [100, 101]. On the other hand, an uncoupling of OGT/OGA levels and altered O-GlcNAcylation has been reported in different diseases, suggesting a role in pathology progression [27, 40, 42, 97, 102]. In Ts2 mice, the relevant reduction in protein O-GlcNAcylation was concomitant with a significant increase of both OGA transcript and protein levels, hinting at a lack of compensation for O-GlcNAc imbalance. Taken together, our data suggest that the overall removal process of O-GlcNAc moiety is markedly increased in young DS animals as an effect of OGA overexpression. We cannot exclude that increased gene dosage, occurring in DS both human and mice, might directly or indirectly drive the overexpression of OGA transcript and protein. However, no interaction between triplicated genes in DS and OGA gene sequence or product has yet been observed, and further studies in that sense are needed.

Molecular pathways regulating the O-GlcNAcylation of proteins include the HBP, an offshoot of the glycolytic flux that integrates several major metabolic pathways into the synthesis of UDP-GlcNAc. Zibrova et al. demonstrated that GFAT1, the first rate-limiting enzyme of the HBP, undergoes negative regulation through increased phosphorylation on Ser243, by the action of AMPK, one of the master sensors of cellular energy. Moreover, AMPK itself was found to be O-GlcNAcylated on several residues, allowing to theorize further feedback mechanisms between these systems [103, 104]. In line with the reduced AMPK activation (Thr172 phosphorylation), GFAT1 inhibitory phosphorylation decreases and a trend of increase is observed for GFAT1 enzyme activity. In addition, HBP is finely regulated by nutrient availability and brain metabolic changes, and many studies demonstrated that DS brain is characterized by the early presence of insulin resistance markers, which precede and contribute the development of AD-like brain damage [16]. We demonstrate in Ts2 mice at 6 months of age that reduced GlcNAc levels couple with the overactivation of insulin signal with no massive defects in glucose uptake. These events suggest not considering the alteration of glucose utilization as a possible negative regulator of the HBP flux. Despite that, prolonged IR stimulation is known to result in IRS-1 inhibition through negative feedback mechanisms [105], though the early alteration of O-GlcNAcylation cycling may interfere with correct insulin signaling in young Ts2 mice, as observed in AD mice [55], paving the way for the onset of insulin resistance condition observed in DS brain [16, 22, 77]. Accordingly, streptozotocin-treated rats showed decreased global O-GlcNAcylation before the appearance of commonly recognized markers of insulin resistance, confirming the relevance of O-GlcNAcylation disturbances in the onset of insulin signaling defects [56].

In the last decade, OGA inhibitors have been proposed as a promising approach to recover the pathological implications of reduced O-GlcNAcylation in neurodegenerative diseases. Growing evidence in murine models of AD have shown that rescuing brain GlcNAc levels, by OGA inhibition, reduces the levels of pathological tau [36, 106–108], limits APP amyloidogenic cleavage and Aβ accumulation [84, 85], boosts mitochondrial activity [28], and promotes the removal of toxic aggregates through macro-autophagy [90]. Data collected in Ts2 mice support a role for dysregulated OGT/OGA cycle in promoting unbalanced O-GlcNAcylation/phosphorylation ratio of tau and APP, thus proposing a role for their aberrant PTMs in favoring the accumulation of their resulting toxic aggregates. Further, previous data in Ts65Dn mice, which share the same genetic background with Ts2, demonstrate altered mitochondrial function and impaired autophagy [77, 109]. In this scenario, Ts2 mice describe a pathological context that strongly encouraged the administration of OGA inhibitors to rescue GlcNAc levels and protect the brain from AD-like neurodegeneration. Yet, our analysis of liver samples in Ts2 mice showed an opposite profile in terms of O-GlcNAcylated/phosphorylated ratio, as observed in diabetes [21, 102, 110], advising for a brain-specific targeting of OGA with the aim to directly subject the brain and avoid possible disturbances in other organs. The intranasal administration of TMG provided positive outcomes in rescuing protein O-GlcNAcylation levels through the inhibition of OGA activity in Ts2 hippocampus. Such effect was followed by the recovery of ^O-GlcNAc^tau deficiency and site-specific reduction of tau phosphorylation. Indeed, upon TMG administration, a reduction on Ser404 phosphorylation was observed while Ser202 and Thr205 were not affected in our model. However, the site-specific effect on tau phosphorylation should not be considered a surprise since compelling evidence indicated that the neuroprotective effect of increased ^O-GlcNAc^tau levels is not necessarily associated with the reduction of each tau phosphorylation sites [85]. In agreement, a previous study by Yang Yu et al., regarding the effects of acute TMG treatment, observed a reduction in tau phosphorylation on Ser404, while an increase in Ser202 was reported together with a time-dependent effect on Thr205 [108]. As a matter of fact, a site-specific reciprocity between O-GlcNAcylation and phosphorylation was described for Ser404 [58, 111]. As regarding APP, TMG has proved to compensate for reduced ^O-GlcNAc^APP levels that characterized DS mice and was able to further reduce its aberrant hyper-phosphorylation. TMG-driven modulation of APP PTMs acquires special relevance in DS neuropathology. Indeed, DS-affected subjects have increased APP gene dosage and overexpress APP, showing early signs of Aβ buildup [13, 112]. Among APP residues that can be O-GlcNAcylated, Thr576 is known to regulate APP trafficking and processing, attenuating Aβ generation [39]. Conversely, increased levels of phosphorylated APP on Thr668 are found in AD brains and seem to facilitate BACE1 cleavage of APP that results in higher Aβ production [81, 82]. In line with this neuroprotective role of APP O-GlcNAcylation, our TMG treatment indicates a redirection of APP toward a non-amyloidogenic processing as suggested by the trend of reduction of β-CTF/α-CTF ratio and of soluble Aβ 1-42. Despite the absence of amyloid plaques, elevation of APP, β-CTF, and soluble Aβ was demonstrated during aging in the hippocampus of the Ts65Dn model [86]. Since increased soluble Aβ oligomers characterized middle-aged Ts65Dn mice [87] only, the absence of increased soluble Aβ 1-42 peptide at 6 months of age in our model was not surprising. However, the modulation of O-GlcNAc APP levels by OGA inhibition suggested a role in the reduction of soluble Aβ 1-42 levels in both Eu and Ts2 mice, as reported elsewhere [73]. In line with this neuroprotective effect, TMG treatment also proved to boost PSD95, Syntaxin 1A, and BDNF expression, whose rescue is commonly associated with improved synaptic plasticity and amelioration of cognitive performances [77, 89]. Although we have shown the positive effects of increasing O-GlcNAcylated levels of both tau and APP PTMs, this is not the only mechanism through which TMG exerts its neuroprotective purposes. A recent study by Zhu et al. strongly indicates that TMG acts in the brain to induce the disposal of toxic aggregates through the enhancement of autophagy [90]. The ability of TMG to modulate autophagy holds particular importance in DS, where defective degradative systems are known to exacerbate neurodegenerative phenotype by preventing proper aggregate clearance [22, 77, 95, 113]. Despite this, Ts2 mice did not show signs of massive autophagy impairment at 6 months of age, and an increase of SQSTM1 was observed, possibly indicating insufficient autolysosomal degradation [114]. TMG treatment demonstrated boosting autophagic flux in our DS mice at both initial and later steps. Indeed, increased levels of Atg7 and Beclin-1 support an effect of TMG treatment at the level of autophagic induction, while increased LC3-II/I ratio and the reduction of SQSTM1 levels suggest a TMG-driven increase of autophagosome maturation and lysosomal degradation in DS mice. The valuable interplay between O-GlcNAcylation and autophagy was also observed in a number of different in vitro and in vivo models, where authors showed how reducing protein O-GlcNAcylation by genetic and pharmacological manipulation of OGT or OGA resulted in increased autophagy [90, 115–117]. In this scenario, our results support that recovering protein O-GlcNAcylation in an altered phenotype such as DS could be an effective strategy to activate autophagic flux.

Notably, TMG treatment was also able to ameliorate the increased 3-NT levels which characterize the pathological alterations occurring in the brain of DS human and mice. Several lines of evidence demonstrated a role for autophagy in the removal of oxidized proteins [77, 96, 118–120] and our data from TMG treatment support this hypothesis. However, the decreased burden of nitrated proteins might also be associated with the reduced formation of toxic tau and APP aggregates and/or with the induction/modulation of antioxidant pathways regulated by the O-GlcNAcylation process.

**Fig. 10:**
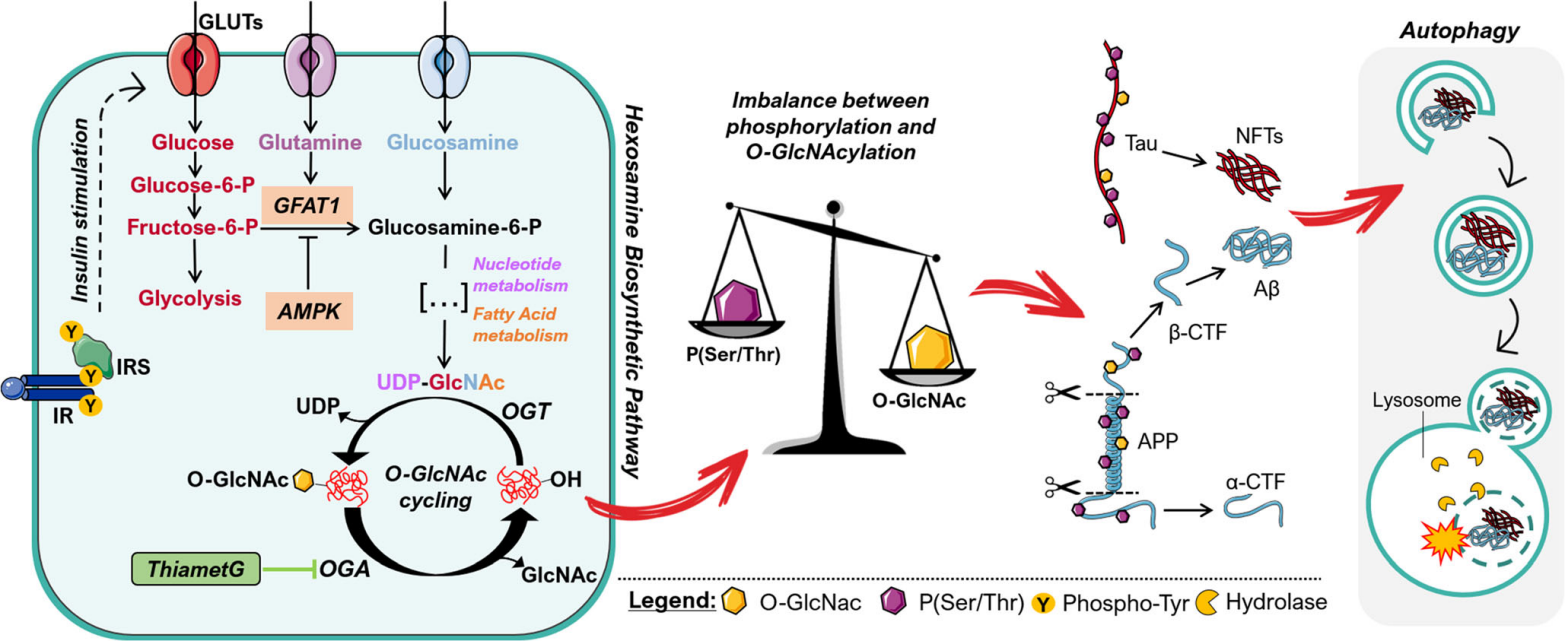
Role of distupted O-GlcNAcylation homeostasis in DS neuropathology. The hexosamine biosynthetic pathway (HBP) is a minor branch of the glycolitic pathway that results in the production of UDP-GlcNAc, the activated substrate for protein O-GlcNAcylation. As the HBP flux integrates molecules from carbohydrate (fructose-6-phosphate), amino acid (glutamine/glucosamine), neucleotide (UTP), and lipid (Acetyl-CoA) metabolism, the production of UDP-GlcNAc is considered a valuable intracellular sensor of cell metabolic status. An early upregulation of the insulin cascade (IR-IRS) is present in our DS murine model, which could reasonably imply an increase in glucose availability. In line with that, the HBP rate-limiting enzyme GFAT1 lack the inhibitory action of the metabolic-sensor kinase AMPK. Therefore, the altered OGT functionality and, mostly, aberrant increase of OGA-driven hydrolysis of O-GlcNAc seem to be the main cause for reduced protein O-GlcNAcylation in our DS model. The loss of protein O-GlcNAcylation is kown to give rise to an aberrant increase of protein phosphorylation, because of the mutual inverse relationship between these two modifications. This process aquires particular relevance when it comes to the post-transaltional modifications of proteins implicated in the DS neurodegenerative process. Indeed, the unbalanced O-GlcNAcylation/phosphorylation ratio of tau is known to promote its aggregated forms (NTFs, neurofibrillary tangles) while the aberrant increase of phosphorylated APP favors its amyloidogenic cleavage that results in the formation of β-CTF and thus, to β-amyloid accumulation. In this scenario, data collected on our model confirm the relevance of O-GlcNAcylation disruption in the appearance of AD-related hallmarks. Furthermore, TMG-mediated inhibition of OGA proved to restore protein O-GlcNAcylation and further exert its neuroprotective effects by boosting autophagic clearance of toxic aggregates and inducing the expression of proteins related to synaptic transmission

Overall, our study supports a pathological role for reduced O-GlcNAcylation in DS mice and poses the dysregulation of OGT/OGA cycle as a central contributor to tau and APP hyperphosphorylation. Inhibiting brain OGA activity, by intranasal TMG administration, recovers total and specific GlcNAc levels of tau and APP, and protects the hippocampus from increased protein nitration in a mechanism involving the induction of autophagy (Fig. 10). In this scenario, the brain-targeted rescue of protein O-GlcNAcylation may represent a valuable therapeutic strategy to ameliorate the early development of AD-like pathology in DS subjects.

## Supplementary Information

ESM 1 (PDF 1224 kb)

ESM 2 (PPTX 6527 kb)

## Abbreviations

3NT: 3 nitrotyrosine
Aβ: amyloid beta
AMPK: AMP-activated protein kinase
APP: amyloid precursor protein
AD: Alzheimer disease
Atg: autophagy related
BACE1: beta secretase 1
BDNF: brain-derived neurotrophic factor
CTF: c-terminal fragment
DG: dentate gyrus
DS: Down syndrome
Eu: euploid
GFAP: glial fibrillary acidic protein
GFAT1: glutamine:fructose-6-phosphate amidotransferase 1
GlcNAc: N-acetylglucosamine
GLUT: glucose transporter
HBP: hexosamine biosynthetic pathway
HNE: hydroxynonenal
IBA1: ionized calcium-binding adapter 1
IR: insulin receptor
IRS1: insulin receptor substrate 1
NFT: neurofibrillary tangles
OGA: (protein)-3-O-(N-acetyl-d-glucosaminyl)-l-serine/threonine N-acetylglucosaminyl hydrolase
OGT: protein O-GlcNAc transferase
PTM: post translational modification
Ser: serine
SQSTM1: sequestosome 1
Thr: threonine
TMG: Thiamet G
Ts2: Ts2Cje
Veh: vehicle
