# Supplementary material for: The Dysregulation of OGT/OGA Cycle Mediates Tau and APP Neuropathology in Down Syndrome
Source: Neurotherapeutics. 2020 Nov 30;18(1):340–63. doi: 10.1007/s13311-020-00978-4 (PMC8116370; doi:10.1007/s13311-020-00978-4)
Supplement: Supplementary file 2 — (PPTX 6527 kb) [file 13311_2020_978_MOESM2_ESM.pptx]

## Slide 1
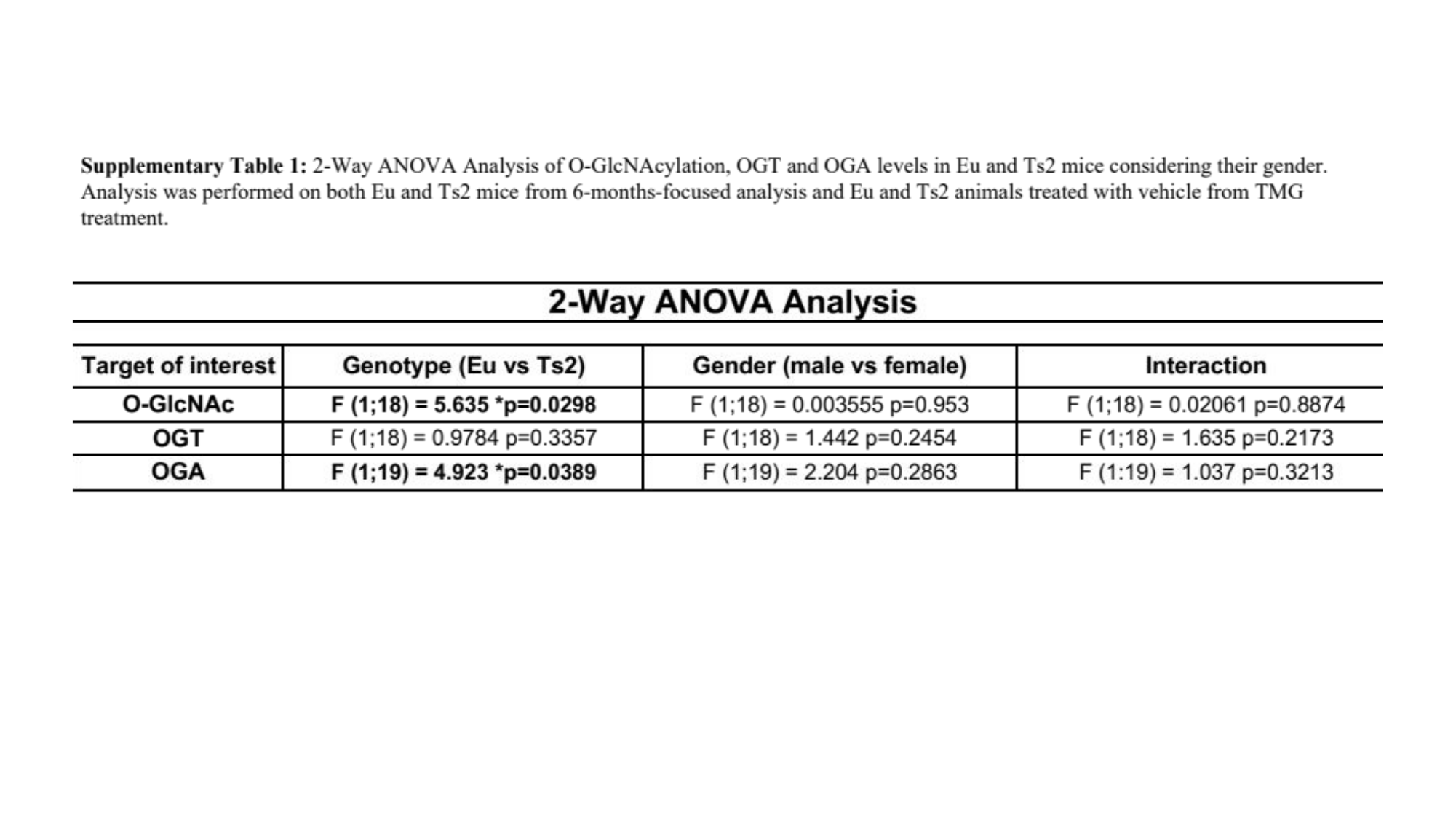

## Slide 2
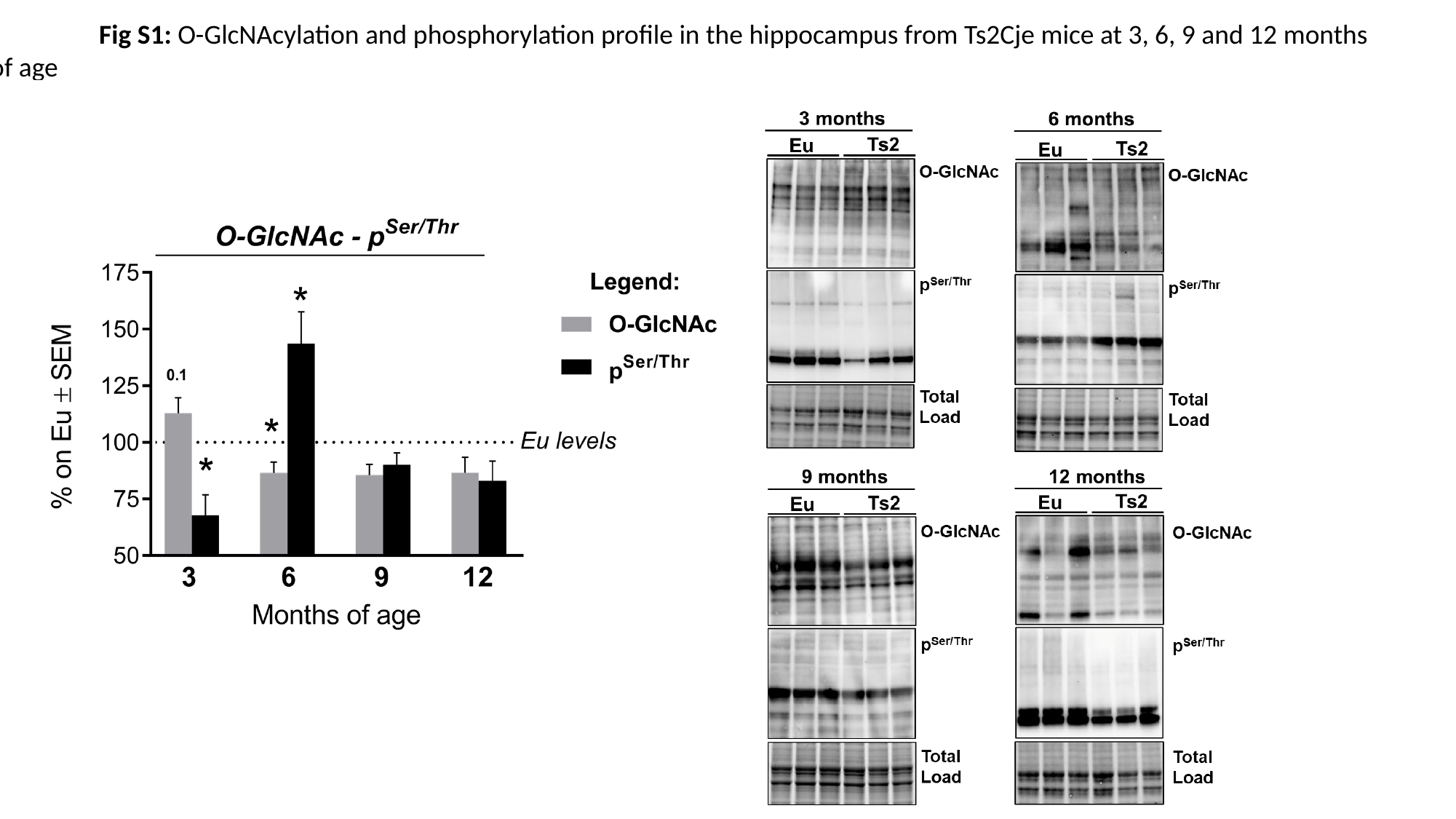

Fig S1: O-GlcNAcylation and phosphorylation profile in the hippocampus from Ts2Cje mice at 3, 6, 9 and 12 months of age
compared to aged-matched Euploids

## Slide 3
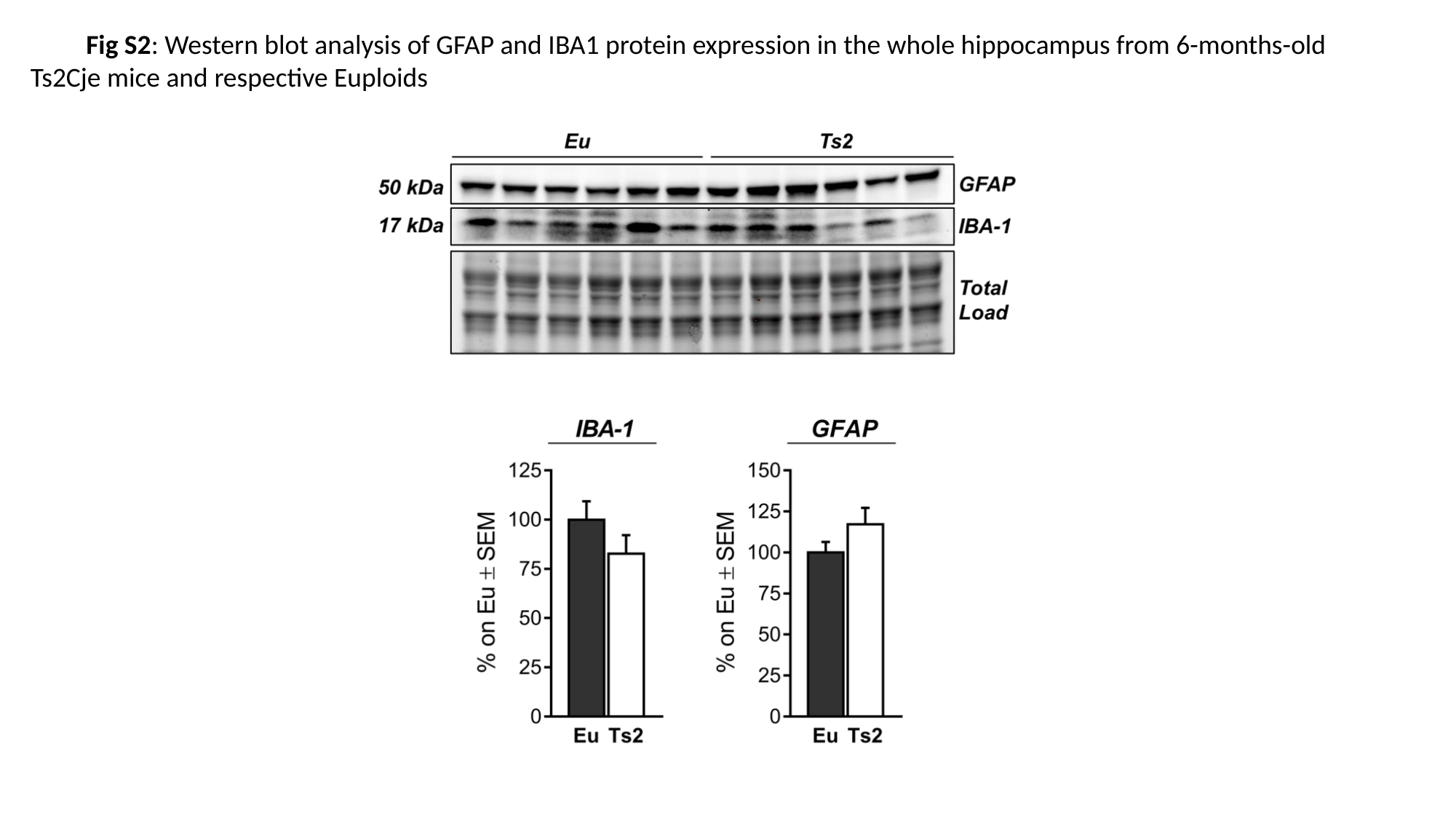

Fig S2: Western blot analysis of GFAP and IBA1 protein expression in the whole hippocampus from 6-months-old Ts2Cje mice and respective Euploids

## Slide 4
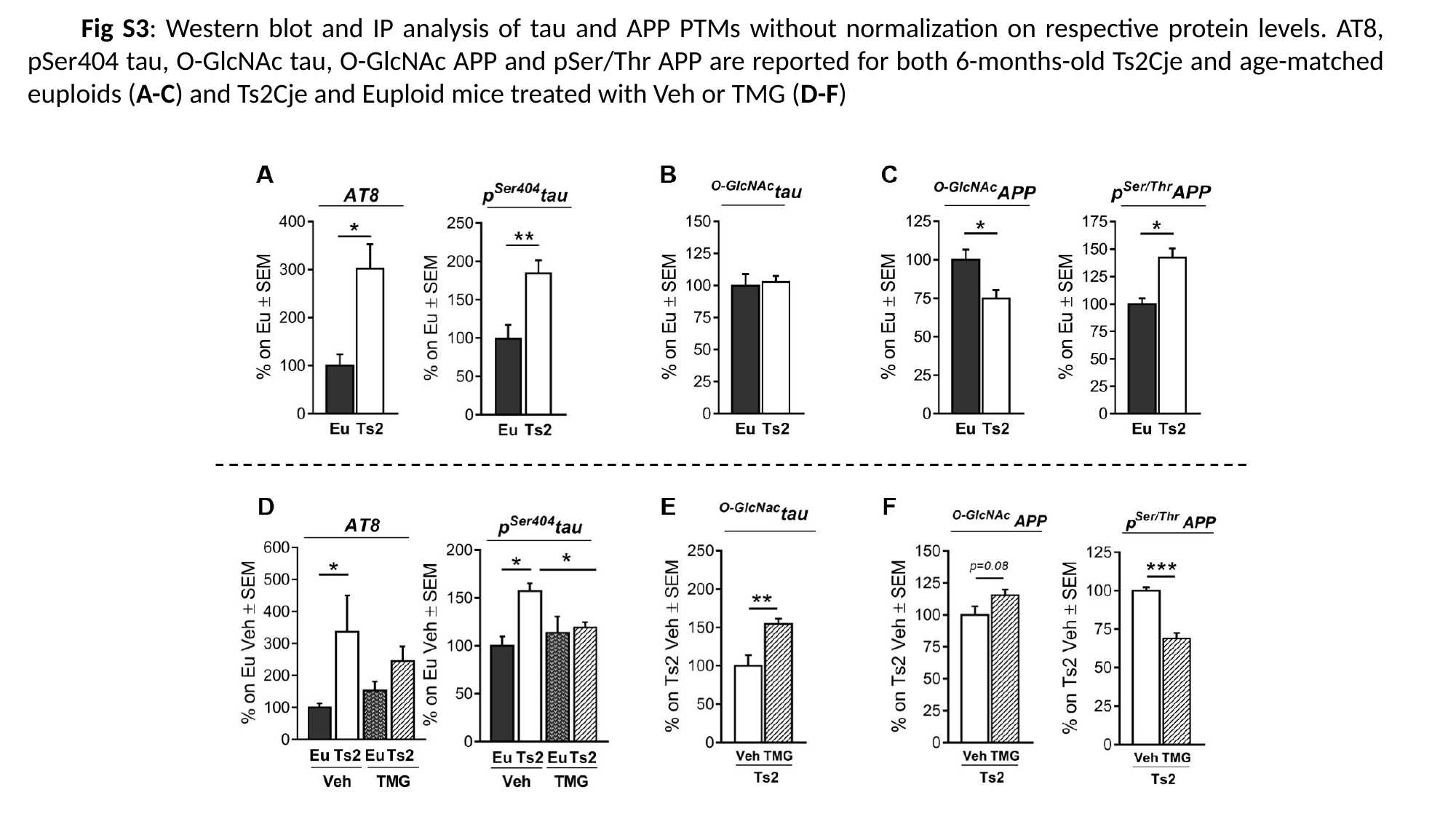

Fig S3: Western blot and IP analysis of tau and APP PTMs without normalization on respective protein levels. AT8, pSer404 tau, O-GlcNAc tau, O-GlcNAc APP and pSer/Thr APP are reported for both 6-months-old Ts2Cje and age-matched euploids (A-C) and Ts2Cje and Euploid mice treated with Veh or TMG (D-F)

## Slide 5
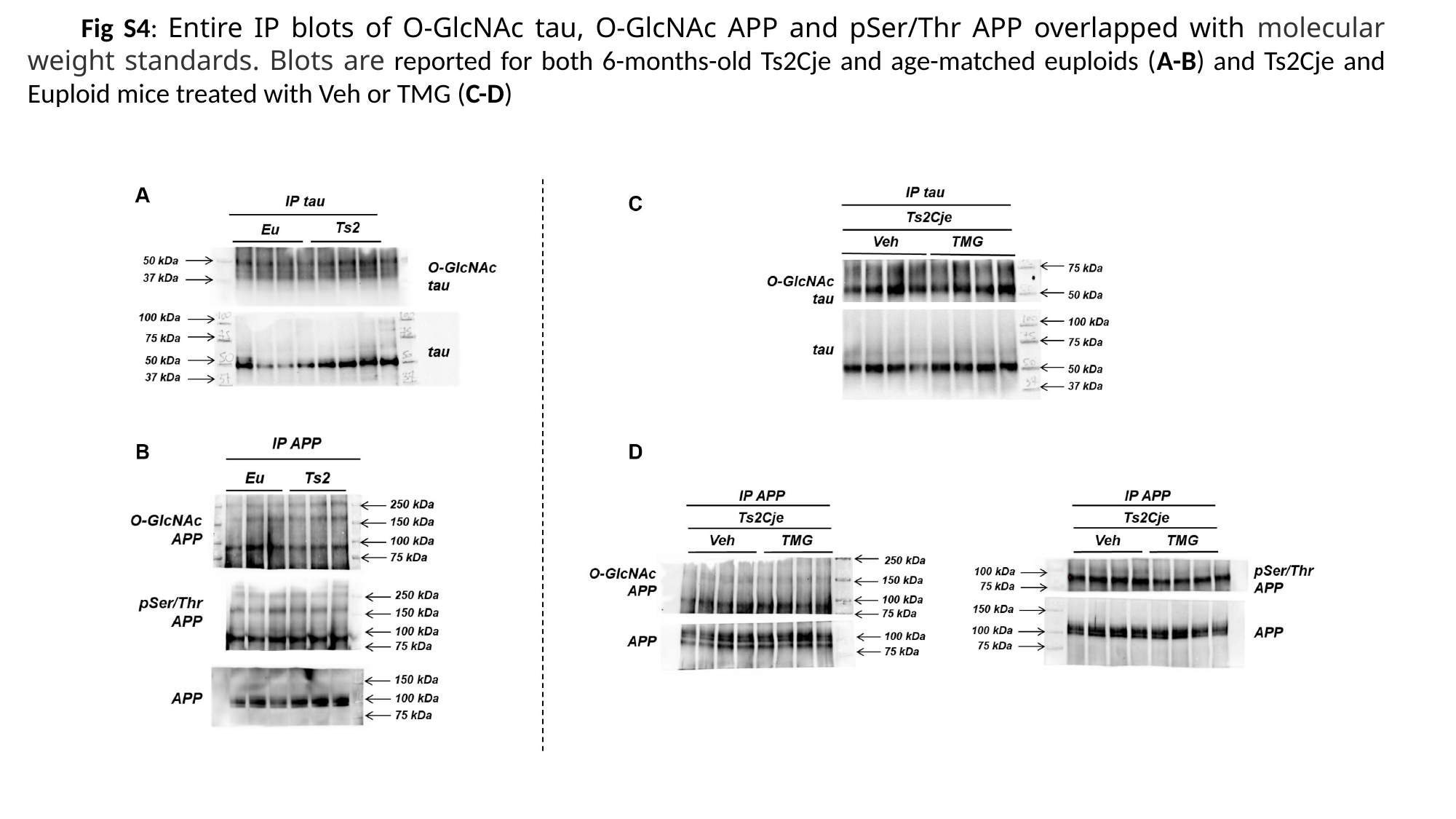

Fig S4: Entire IP blots of O-GlcNAc tau, O-GlcNAc APP and pSer/Thr APP overlapped with molecular weight standards. Blots are reported for both 6-months-old Ts2Cje and age-matched euploids (A-B) and Ts2Cje and Euploid mice treated with Veh or TMG (C-D)

## Slide 6
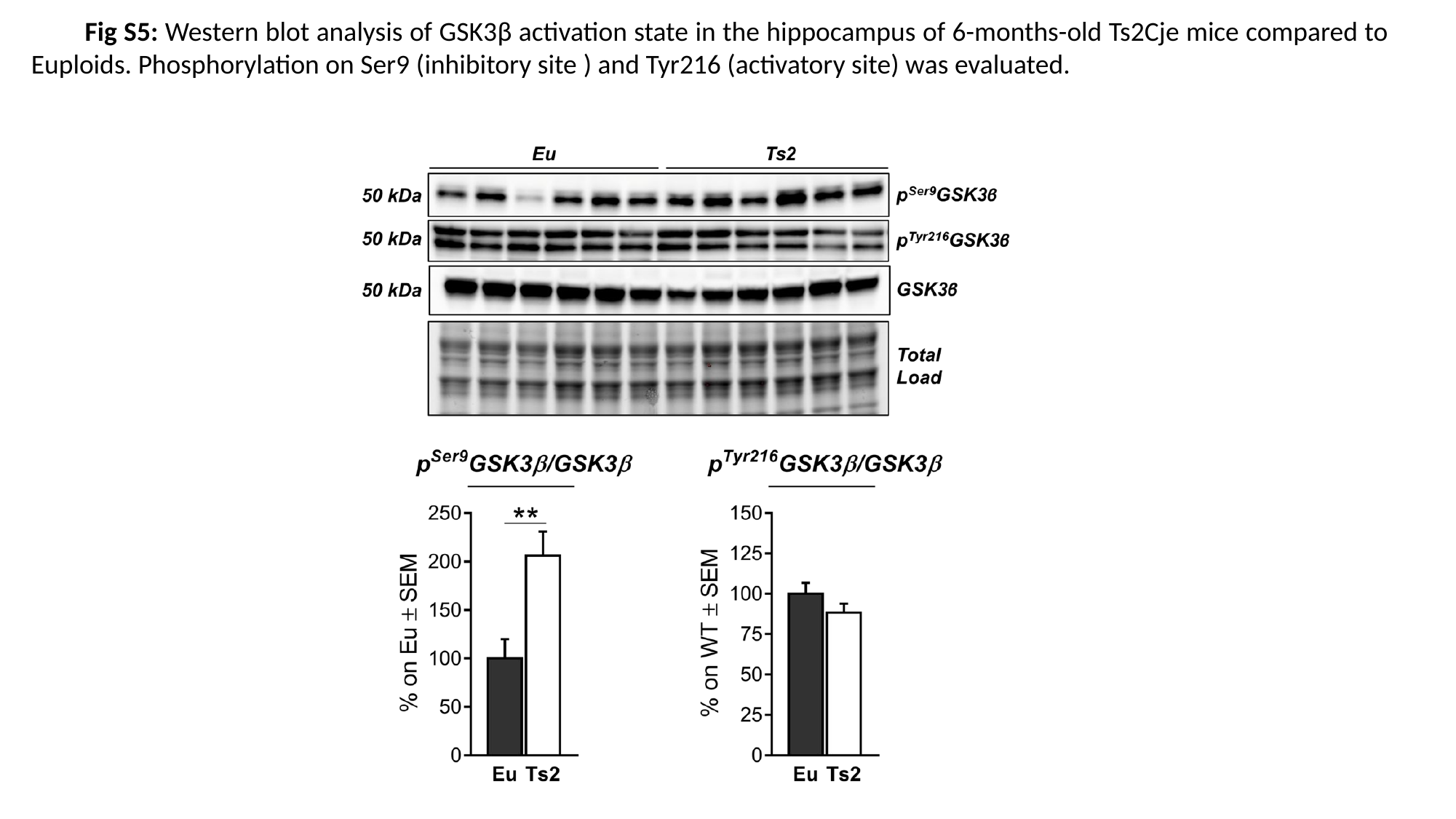

Fig S5: Western blot analysis of GSK3β activation state in the hippocampus of 6-months-old Ts2Cje mice compared to Euploids. Phosphorylation on Ser9 (inhibitory site ) and Tyr216 (activatory site) was evaluated.

## Slide 7
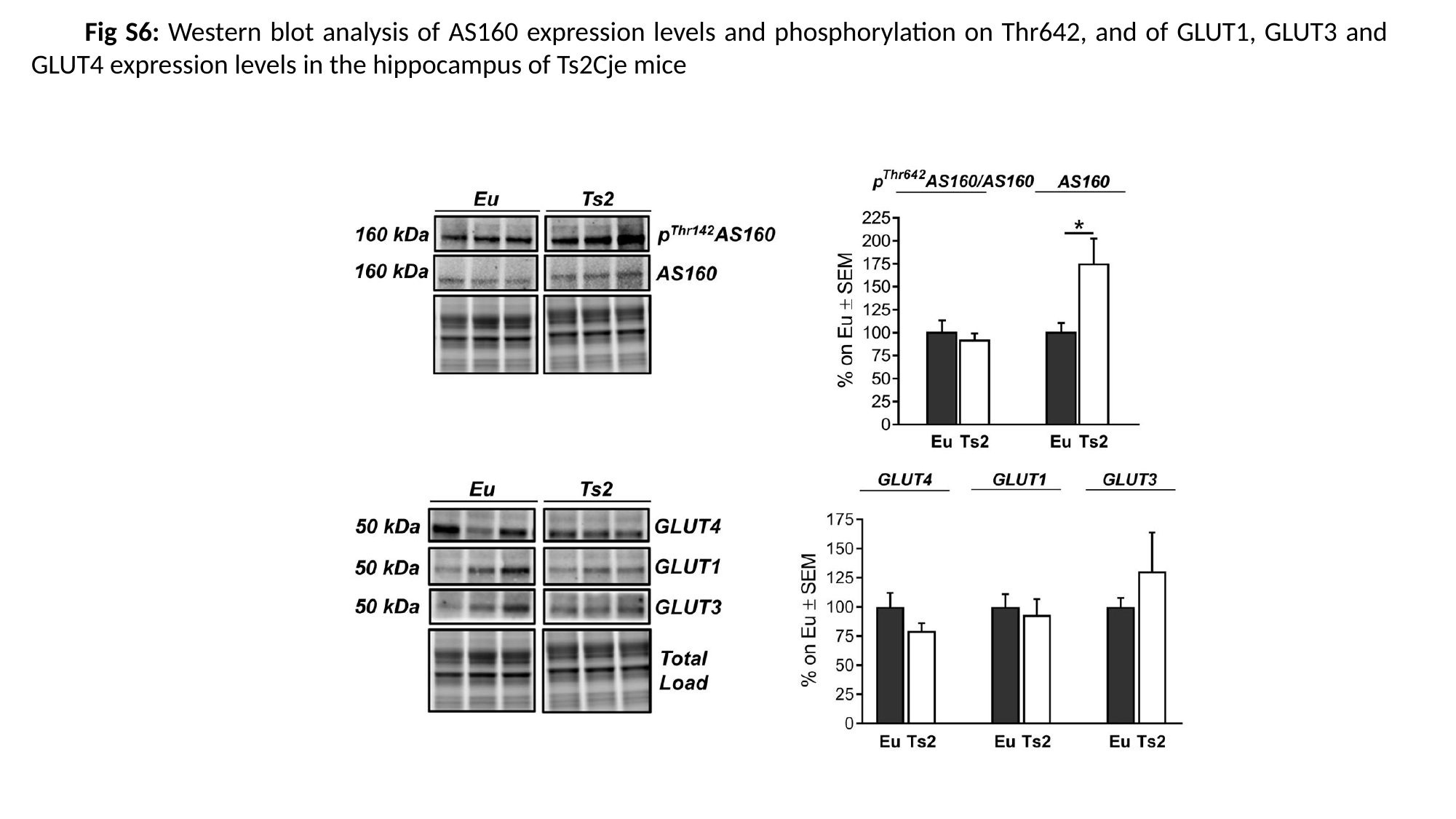

Fig S6: Western blot analysis of AS160 expression levels and phosphorylation on Thr642, and of GLUT1, GLUT3 and GLUT4 expression levels in the hippocampus of Ts2Cje mice

## Slide 8
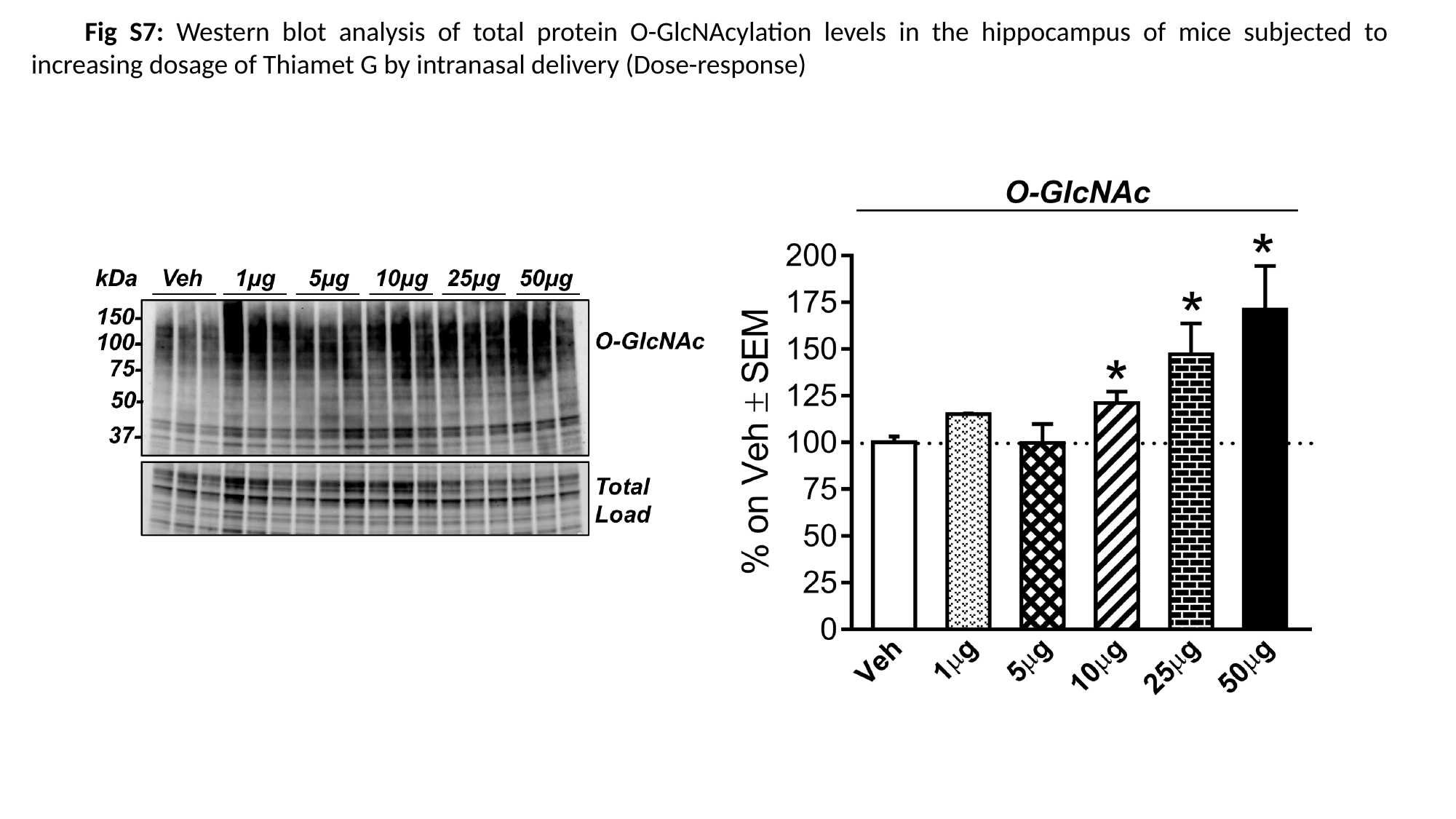

Fig S7: Western blot analysis of total protein O-GlcNAcylation levels in the hippocampus of mice subjected to increasing dosage of Thiamet G by intranasal delivery (Dose-response)

## Slide 9
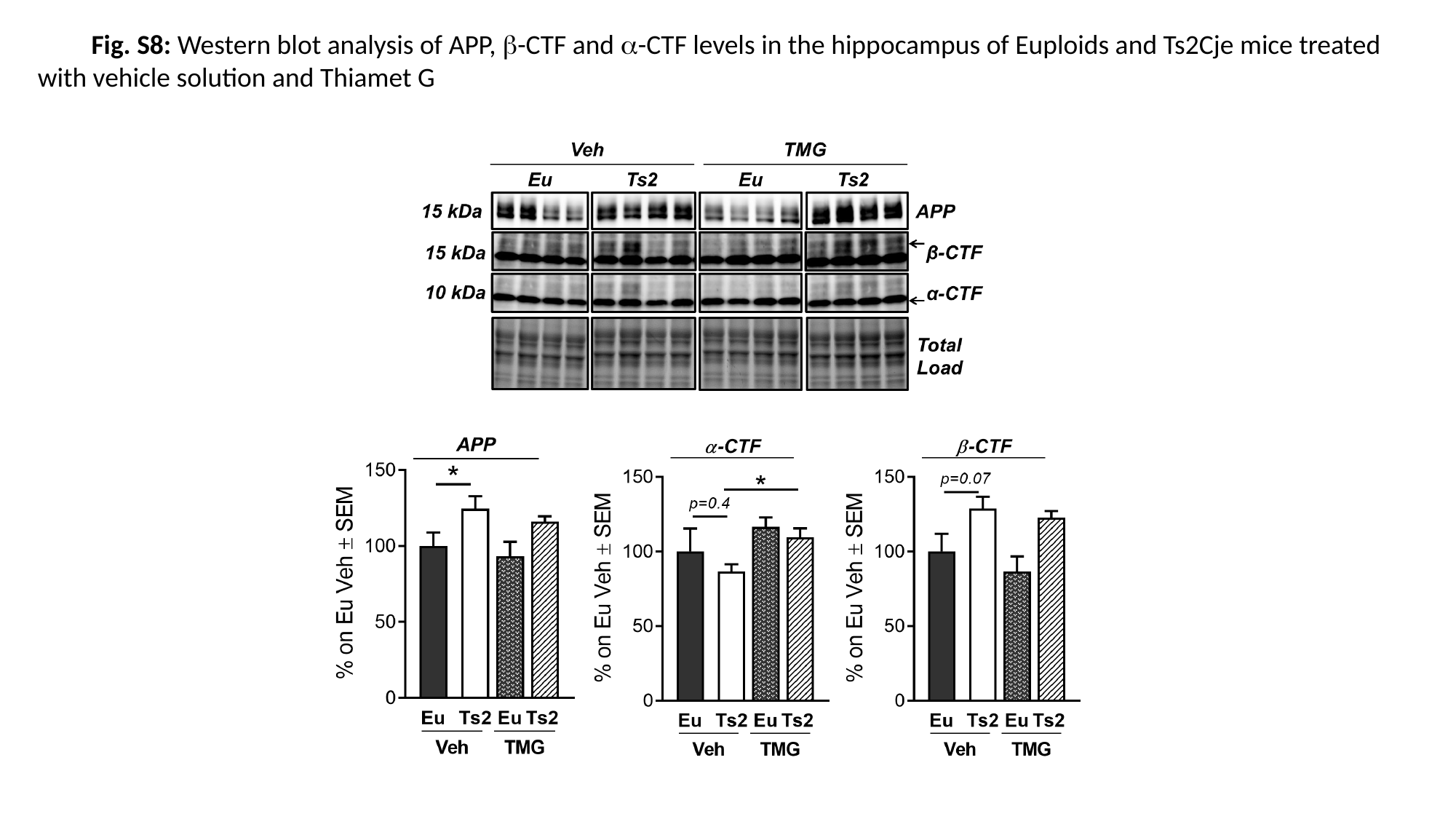

Fig. S8: Western blot analysis of APP, b-CTF and a-CTF levels in the hippocampus of Euploids and Ts2Cje mice treated with vehicle solution and Thiamet G

## Slide 10
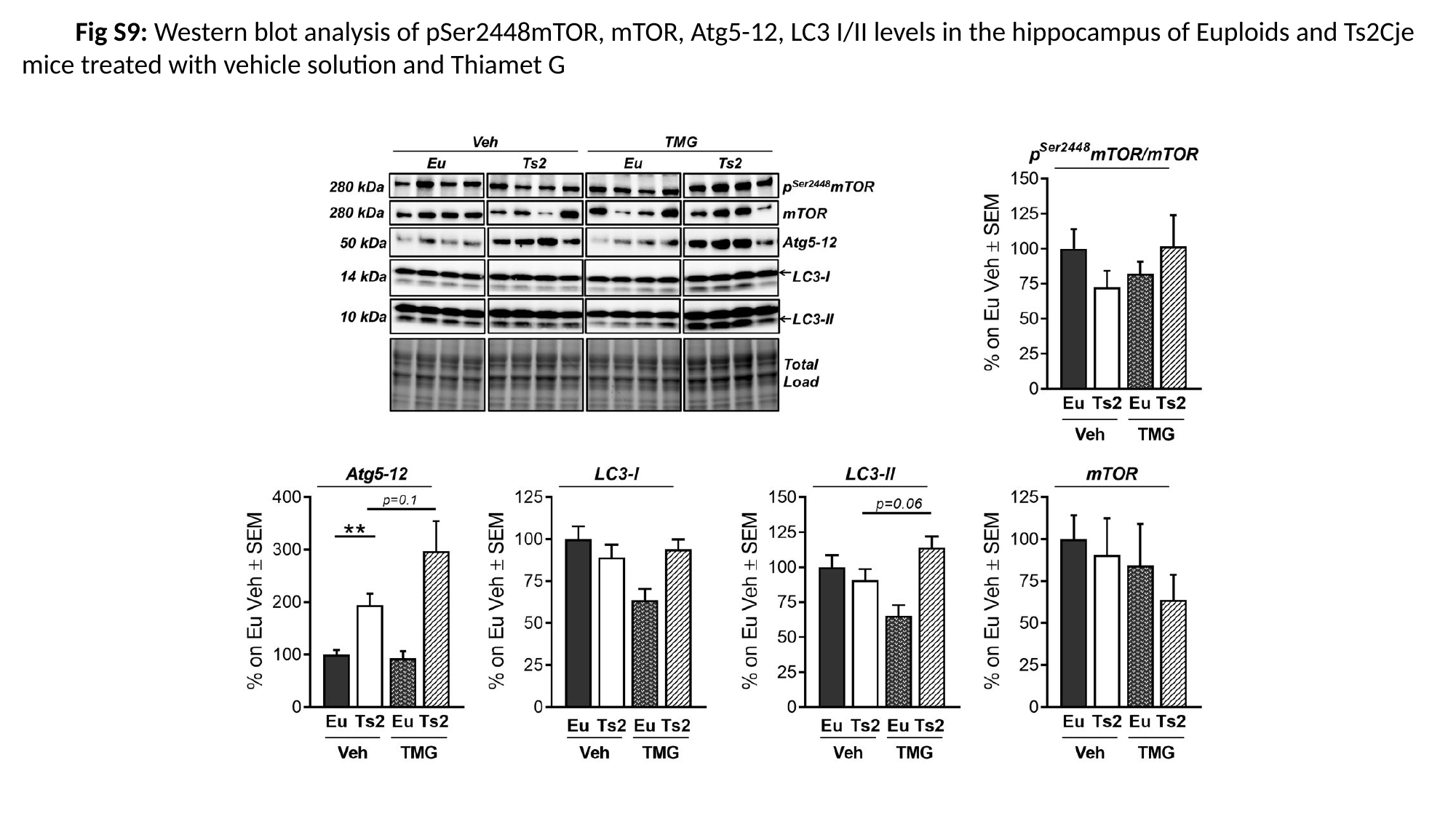

Fig S9: Western blot analysis of pSer2448mTOR, mTOR, Atg5-12, LC3 I/II levels in the hippocampus of Euploids and Ts2Cje mice treated with vehicle solution and Thiamet G
